# Derivation of feeder-free human extended pluripotent stem cells

**DOI:** 10.1016/j.stemcr.2021.07.019

**Published:** 2021-09-14

**Authors:** Ran Zheng, Ting Geng, Dan-Ya Wu, Tianzhe Zhang, Hai-Nan He, Hai-Ning Du, Donghui Zhang, Yi-Liang Miao, Wei Jiang

## Main text

(Stem Cell Reports *16*, 1686–1696; July 13, 2021)

In the fifth paragraph of the results section in the original version of our manuscript, the citation for Figure 1I at the end of the sentence that read, “We checked our data, and indeed, nine of those ten eight-cell-specific genes showed higher expression in ffEPSCs compared with parental ESCs (KMT5A highly expressed in both ESC and ffEPSC samples) (Figure 1I)” should have read “Figure S1H.” Additionally, the originally published version of Figure 1I contained an incorrect panel label, “8 Cell-specific genes (*Stirparo et al., Development, 2018*),” which has now been corrected to “Early Embryo Specific Genes.” The citation and figure have been replaced in the online version of our manuscript, and both the original and corrected versions of Figure 1 appear below.

Additionally, Figure S1H (along with the panel label “8-cell-specific markers (*Stirparo et al., Development, 2018*)”) was previously missing from the originally published supplemental information, and it has now been added. Both the original and corrected versions of Figure S1 appear below as well.

**Figure:**
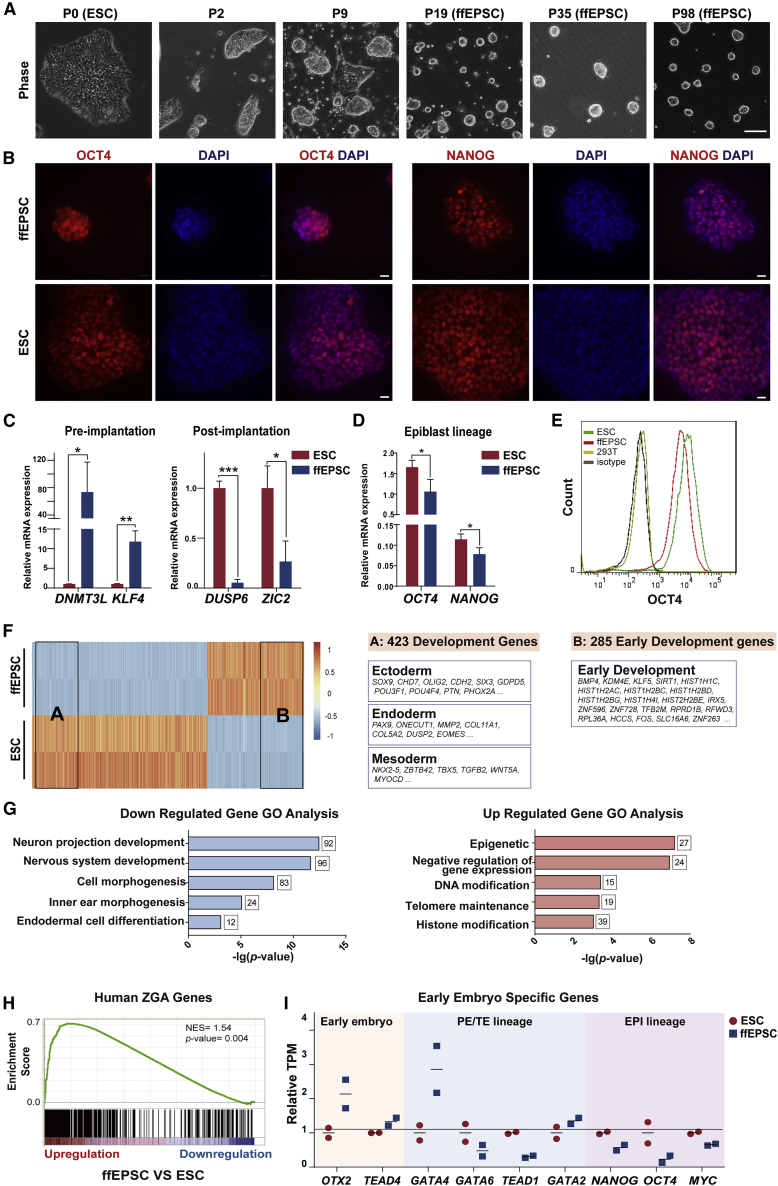
Figure 1. Generation of human ffEPSCs from ESCs under feeder-free conditions (corrected)

**Figure:**
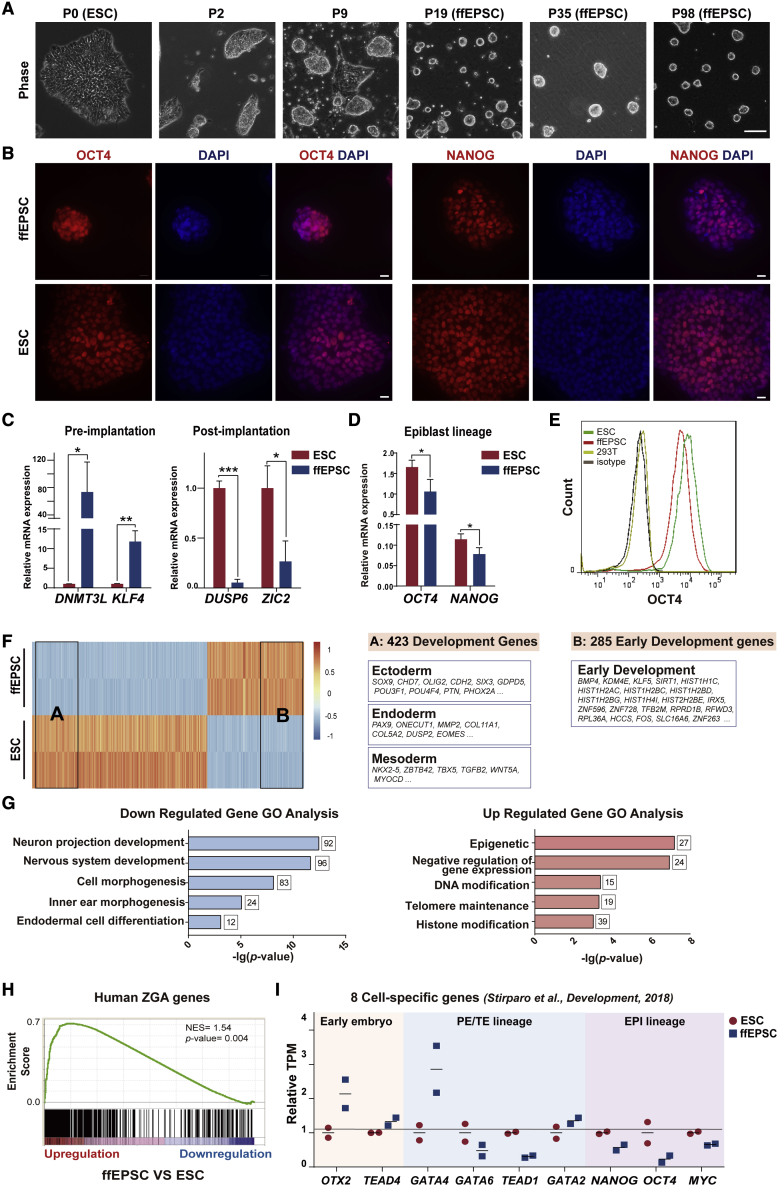
Figure 1. Generation of human ffEPSCs from ESCs under feeder-free conditions (original)

**Figure:**
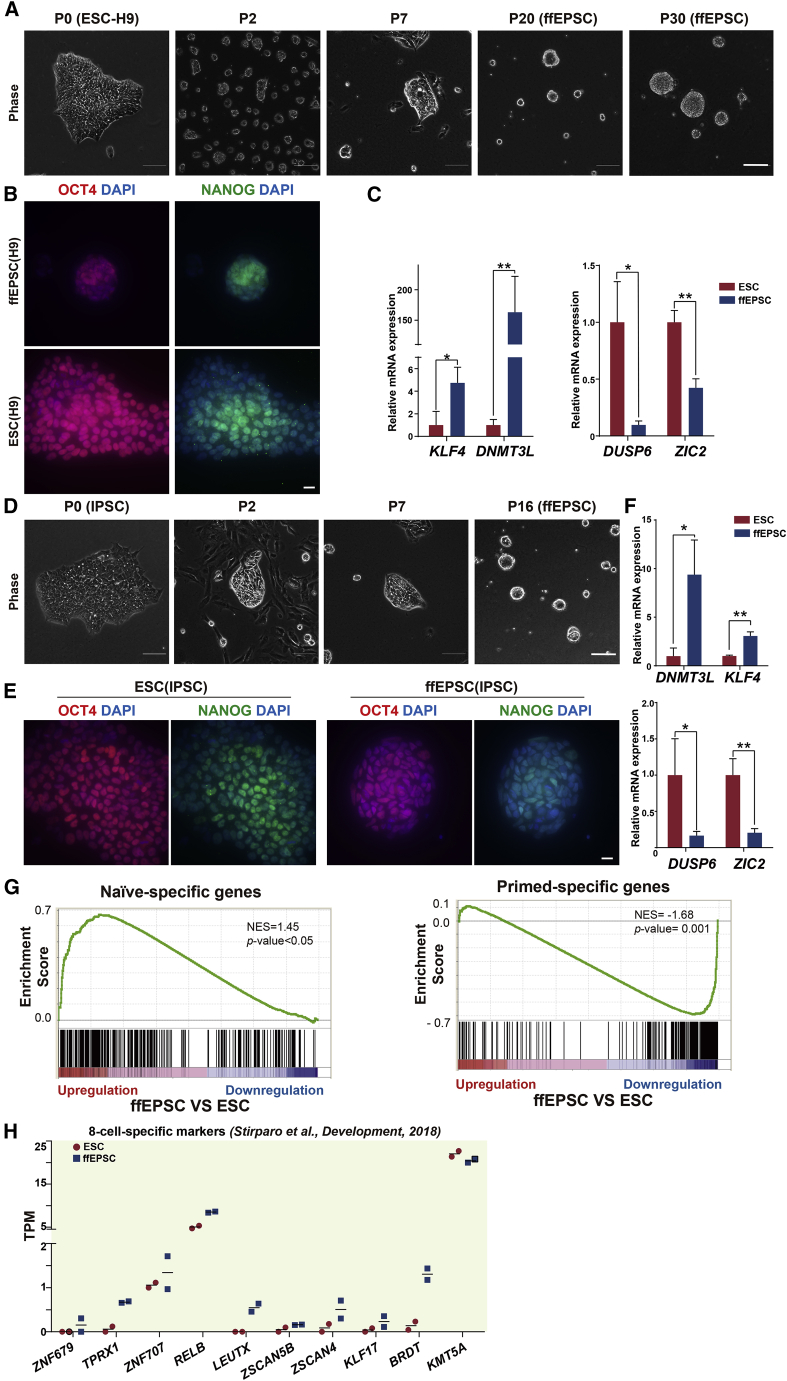
Figure S1. Generation of human ffEPSCs from H9 or iPSCs under feeder-free condition (corrected)

**Figure:**
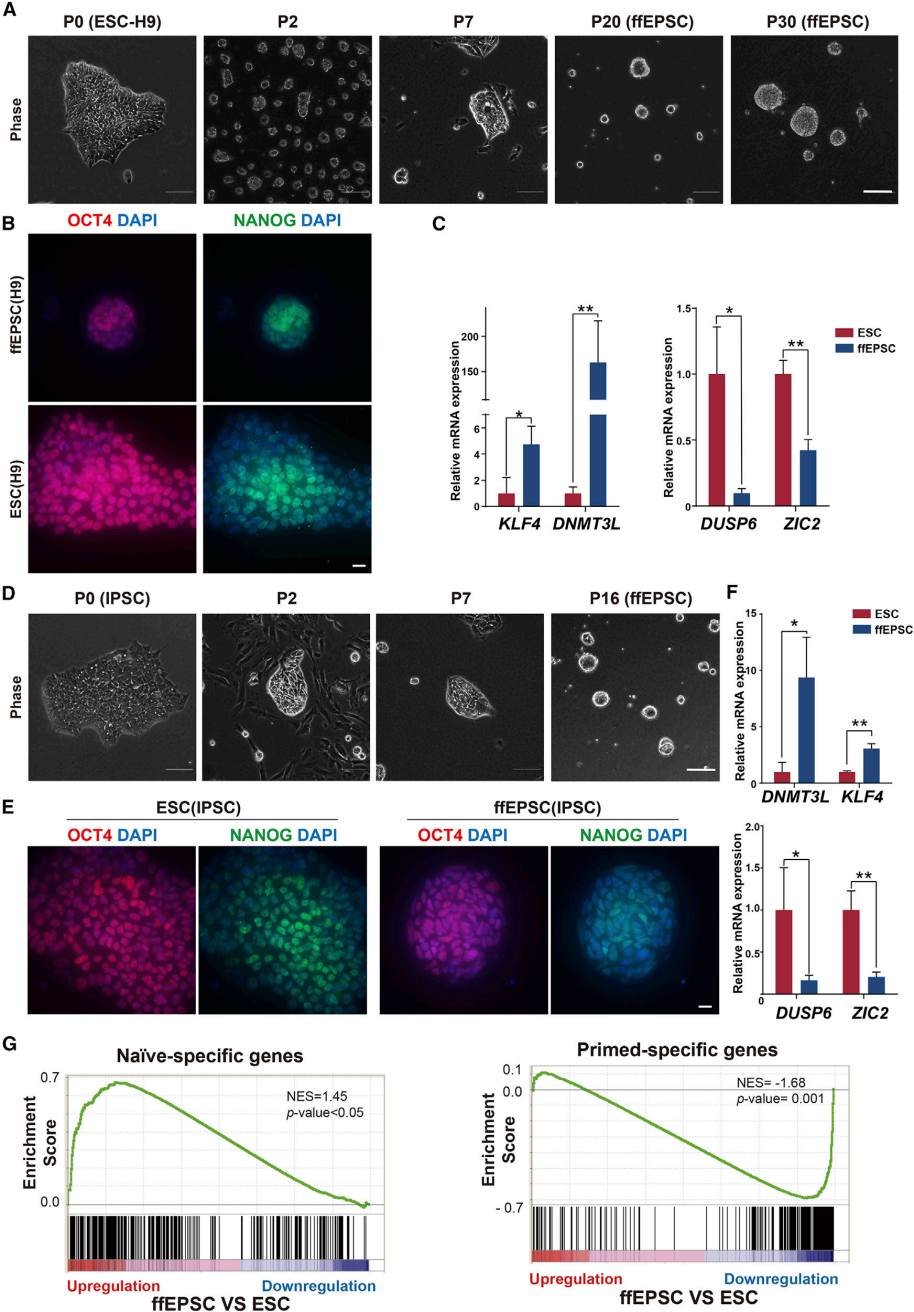
Figure S1. Generation of human ffEPSCs from H9 or iPSCs under feeder-free condition (original)

