# Supplementary material for: Derivation of feeder-free human extended pluripotent stem cells
Source: Stem Cell Reports. 2021 Jul 1;16(7):1686–96. doi: 10.1016/j.stemcr.2021.06.001 (PMC8282469; doi:10.1016/j.stemcr.2021.06.001)
Supplement: Document S1. Supplemental experimental procedure and Figures S1–S3 [file mmc1.pdf]

Stem Cell Reports, Volume *16*

Supplemental Information

Derivation of feeder-free human extended pluripotent stem cells

Ran Zheng, Ting Geng, Dan-Ya Wu, Tianzhe Zhang, Hai-Nan He, Hai-Ning Du, Donghui Zhang, Yi-Liang Miao, and Wei Jiang

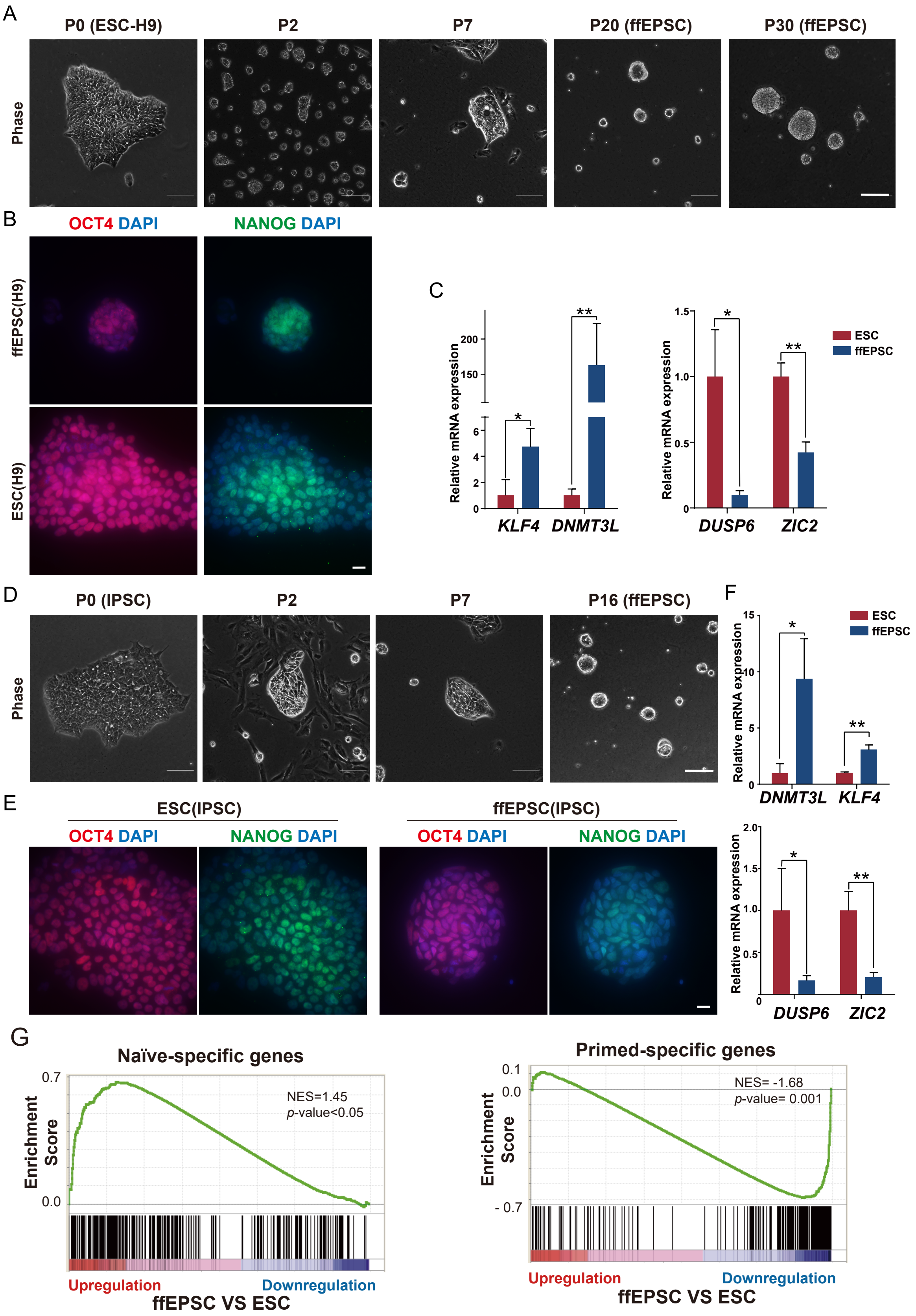
A
P0 (ESC-H9)
P2
P7
P20 (ffEPSC)
P30 (ffEPSC)
Phase
B
OCT4 DAPI
NANOG DAPI
ffEPSC(H9)
ESC(H9)
C
Relative mRNA expression
KLF4
DNMT3L
DUSP6
ZIC2
ESC
ffEPSC
D
P0 (IPSC)
P2
P7
P16 (ffEPSC)
Phase
E
ESC(IPSC)
ffEPSC(IPSC)
OCT4 DAPI
NANOG DAPI
OCT4 DAPI
NANOG DAPI
F
Relative mRNA expression
ESC
ffEPSC
DNMT3L
KLF4
DUSP6
ZIC2
G
Naïve-specific genes
Enrichment Score
NES=1.45
p-value<0.05
Upregulation
Downregulation
ffEPSC VS ESC
Primed-specific genes
Enrichment Score
NES= -1.68
p-value= 0.001
Upregulation
Downregulation
ffEPSC VS ESC

Figure S1. Generation of human ffEPSCs from H9 or iPSCs under feeder-free condition.

A. The morphology of cells during the transition of ESC H9 into ffEPSCs. Scale bars = 100 μm.

B. H9-derived ffEPSCs showed positive staining of NANOG and OCT4. Scale bars = 20 μm.

C. Expression patterns of pre-implantation genes and post-implantation genes in ffEPSCs compared to H9-ESCs (three independent experiments).

D. The morphology of cells during the transition of human iPSCs into ffEPSCs. Scale bars = 100 μm.

E. human iPSC-derived ffEPSCs showed positive staining of NANOG and OCT4. Scale bars = 20 μm.

F. Expression patterns of pre-implantation genes and post-implantation genes in ffEPSCs compared to iPSCs (three independent experiments)

G. GSEA analysis showed the expression pattern of naive-specific genes and primed-specific genes in human ffEPSCs and ESCs.

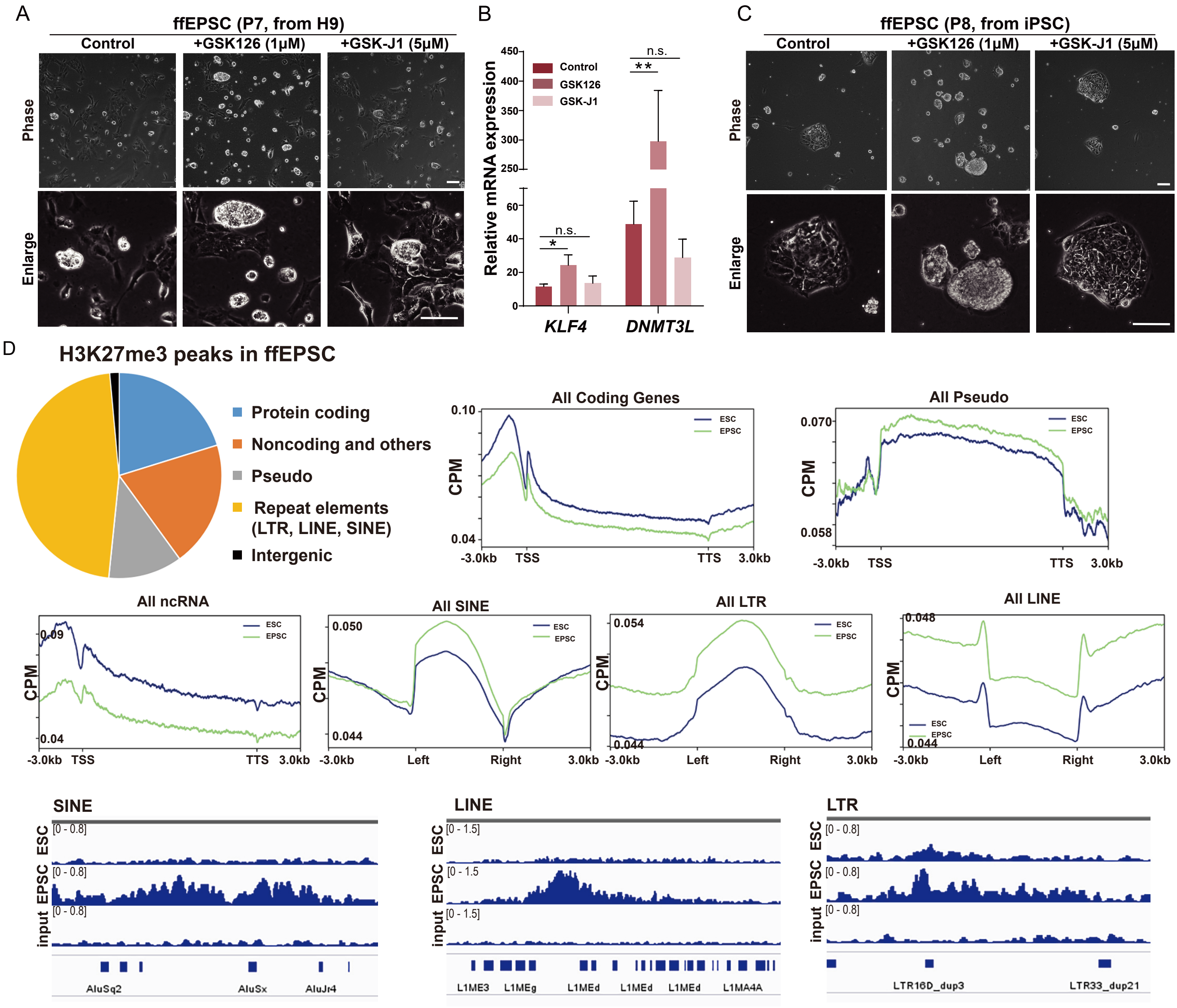
A
ffEPSC (P7, from H9)
Control
+GSK126 (1μM)
+GSK-J1 (5μM)
Phase
Enlarge
B
Relative mRNA expression
Control
GSK126
GSK-J1
n.s.
**
*
KLF4
DNMT3L
C
ffEPSC (P8, from iPSC)
Control
+GSK126 (1μM)
+GSK-J1 (5μM)
Phase
Enlarge
D
H3K27me3 peaks in ffEPSC
Protein coding
Noncoding and others
Pseudo
Repeat elements (LTR, LINE, SINE)
Intergenic
All Coding Genes
All Pseudo
All ncRNA
All SINE
All LTR
All LINE
CPM
ESC
EPSC
-3.0kb
TSS
TTS
3.0kb
Left
Right
SINE
LINE
LTR
ESC
EPSC
input
[0 - 0.8]
[0 - 1.5]
AluSq2
AluSx
AluJr4
L1ME3
L1MEg
L1MEd
L1MA4A
LTR16D_dup3
LTR33_dup21

Figure S2. GSK126 facilitated iPSC-derived human ffEPSC transition.

A, Morphology of H9-derived ffEPSCs cultured with different chemical inhibitors GSK126 and GSK-J1. Scale bars = 100 μm.

B, Gene expression levels of ffEPSCs cultured with GSK-126, GSK-J1 or vehicle control (three independent experiments).

C. Morphology of iPSC-derived ffEPSCs cultured with different chemical inhibitors GSK126 and GSK-J1. Scale bars = 100 μm.

D. Aggregated genomic profiles of H3K27me3 ChIP-Seq around the pcGENE, psGENE, ncRNA, LINE, SINE, LTR and peak distribution.

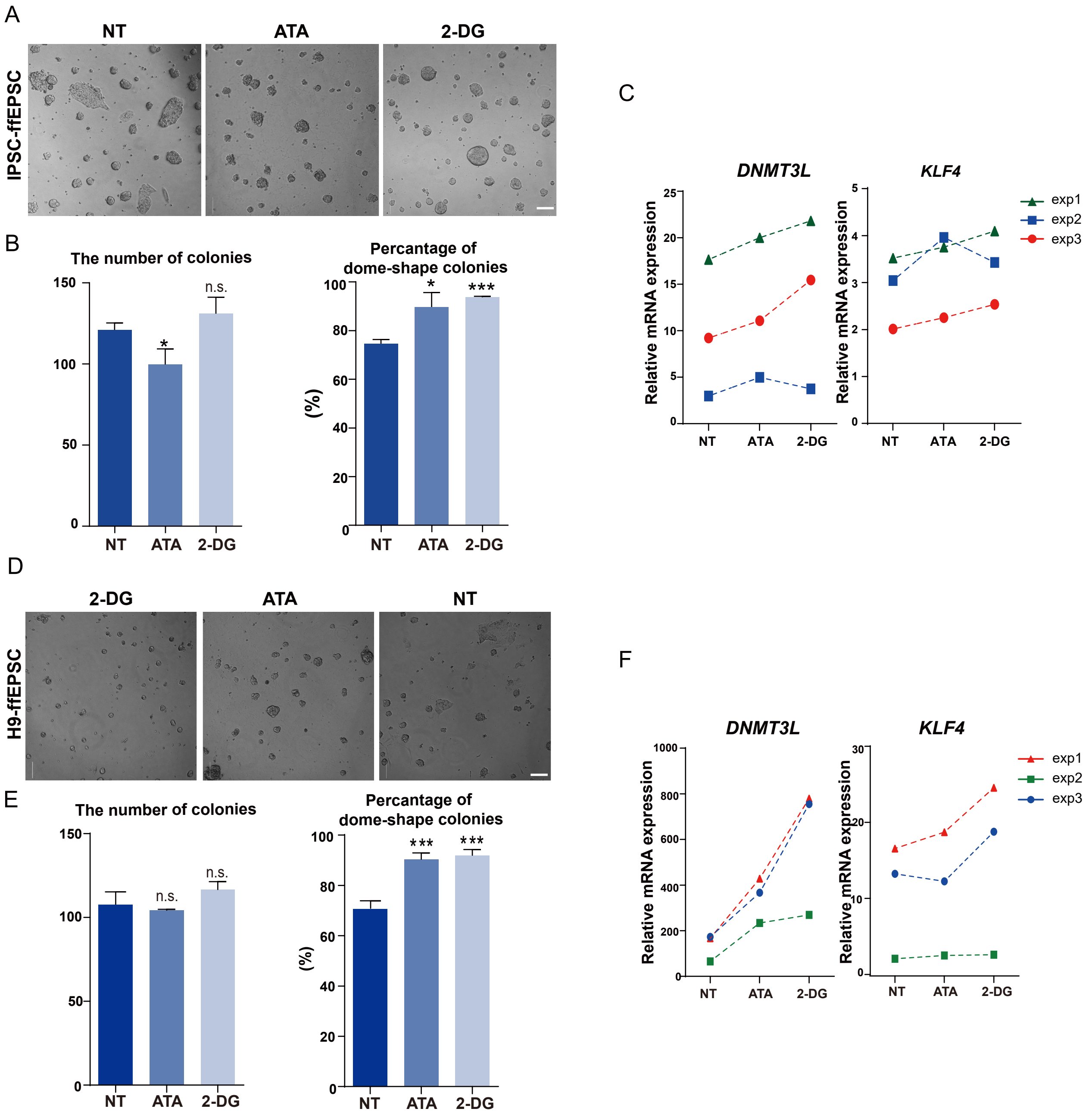

A
NT
ATA
2-DG
IPSC-ffEPSC
B
The number of colonies
Percantage of dome-shape colonies
(%)
n.s.
*
***
C
DNMT3L
KLF4
Relative mRNA expression
exp1
exp2
exp3
D
H9-ffEPSC
E
F

Figure S3. Glycolysis inhibitor 2-DG and ATA facilitated H9 or iPSC-derived human ffEPSC maintenance.

A. Morphology of H9-derived ffEPSCs cultured with different chemical inhibitors 2-DG and ATA. Scale bars = 100 μm.
B. quantitative number and percentage of dome-shaped colonies of H9-derived ffEPSCs cultured with different chemical inhibitors 2-DG and ATA (three independent experiments).
C, RT-qPCR analysis of pre-implantation genes after treatment with glycolytic inhibitors. Results from three independent experiments were shown.
D. Morphology of iPSC-derived ffEPSCs cultured with different chemical inhibitors 2-DG and ATA. Scale bars = 100 μm.
E. quantitative number and percentage of dome-shaped colonies of iPSC-derived ffEPSCs cultured with different chemical inhibitors 2-DG and ATA (three independent experiments).
F, RT-qPCR analysis of pre-implantation genes after treatment with glycolytic inhibitors. Results from three independent experiments were shown.

Supplemental information：

Table S1: RNA-seq data of EPSCs and ESCs

Experimental Procedures

Immunofluorescence staining

Cells were grown on 24-well plates and washed by DPBS before fixed with 4% paraformaldehyde for 20 minutes at room temperature. Fixed cells were blocked with PBS containing 10% donkey serum and 0.3% Triton X-100 for 2 hours at room temperature. Then cells were incubated in the blocking buffer with diluted primary antibodies at 4°C overnight or at room temperature for 2 hours. The cells were further incubated in the blocking buffer with diluted second antibodies at room temperature for 2 hours after three times' washing with DPBS. Nuclei were counterstained with DAPI (1:10000, Thermo-Fisher).

Mouse embryos were collected to 96-well plates with a round bottom, fixed with 4% paraformaldehyde for overnight at 4°C, and then permeated in PBS containing 0.5% Triton X-100 for 30 minutes and blocked with 10% donkey serum in PBS containing 0.3% Triton X-100 for 2 hours at room temperature. Three times' washing with DPBS containing 0.01% Triton X-100 and 0.1% tween-20 was applied before every step. Embryos were incubated in the blocking buffer with diluted primary antibodies at 4°C overnight, and then further incubated with diluted second antibodies at room temperature for 1 hour after washing. At last, embryos were put in mounting medium with DAPI for image capture under the Zeiss LSM880 microscope.

The antibodies used were listed as follows: Anti-NANOG mouse IgG (SantaCruz, #SC-293121, 1:200), Anti-OCT4 mouse IgG (SantaCruz, #SC-5279, 1:200), Anti-OCT4 rabbit IgG (Cell Signaling Technologies, #2750, 1:200), Anti-GATA3 rabbit IgG (Cell Signaling Technologies, #5852, 1:200), Anti-GATA6 rabbit IgG (Cell Signaling Technologies, #5851, 1:200), Anti-OCT3/4 rabbit IgG (BD, #611203, 1:200,). Anti-aromatase rabbit IgG (ABclonal, #A12684, 1:100), Anti-MCT1 rabbit IgG (ABclonal, #A3013, 1:100), Anti-CDX2 rabbit IgG (ZSGB-bio, #ZA0520, 1:200), Donkey-Anti-Mouse-TRITC (Jackson immuno Research, #715-025-150, 1:200), Donkey-Anti-

Mouse-488 (Jackson immuno Research, #715-545-150, 1:200), Donkey-Anti-Rabbit-TRITC (Jackson immuno Research, #711-025-152, 1:200), Donkey-Anti-Rabbit-FITC (Jackson immuno Research, #711-095-152, 1:200).

Flow cytometry

Human ESCs were dissociated with accutase and ffEPSCs were dissociated with TrypLE, to generate single-cell suspension. Intracellular flow cytometry was operated with the Transcription Factor Buffer Set (BD Biosciences). Cells were stained with primary and then secondary antibodies diluted with 2% FBS in PBS containing 0.3% Triton X-100. Data were collected on a FACS Celesta flow cytometer (Becton Dickinson) and analyzed using FlowJo.

Western blot

Cell numbers were counted. Cells were lysed with 1X loading buffer, boiled for 10 minutes at 100°C, Proteins lysates were separated by 15% sodium dodecyl sulfate–poly-acrylamide gel electrophoresis and transferred onto a nitrocellulose membrane (Millipore). The membrane was blocked with 5% BSA in TBS buffer with 0.1% Tween-20 and incubated with antibody against H3K27me3 (1:1000, Cell Signaling Technology, #9733S), H3K4me3 (1:1000, Abclonal, #WH102597), H3K36me3 (1:3000, ABclonal, #A2366), H3K36me2 (1:3000, ABclonal, #A2365), H3K36me (1:3000, ABclonal, #A2364), H3K79me2 (1:3000, ABclonal, #A2368), H3K36Ac (1:500, ABclonal, #A16077) or H3 (1:3000, Proteintech, #17168-1-AP) overnight at 4°C. Then, the membrane was washed and incubated with secondary antibodies at room temperature for 2 hours. After washing, the membrane was visualized with SuperSignal® West Pico Chemiluminescent Substrate (ThermoFisher).

RT-qPCR

Total RNA was extracted with the HiPure Total RNA Mini Kit (Magen). 1-2 μg of total RNA was reversely transcribed into complementary DNA with 5×qRT super Mix.

qPCR was done in duplicated with 2×SYBR Green qPCR Master Mix (Bio-Rad). Glyceraldehyde-3-phosphate dehydrogenase (GAPDH) was used as an endogenous housekeeping control. Student's t-test (two-tailed, equal variance) was performed to obtain p-values for RT-qPCR experiments. Primer sequences are listed as follows: *NANOG* (CCCCAGCCTTTACTCTTCCTA, CCAGGTTGAATTGTTCCAGGTC); *OCT4* (CAAAGCAGAAACCCTCGTGC, TCTCACTCGGTTCTCGATACTG); *KLF4* (ACCCACACAGGTGAGAAACC, ATGCTCGGTCGCATTTTTGG); *DNMT3L* (CGCCCCATGTAAGGACAAGT, ATCGGGTGCAATCAGGGTTT); *ZIC2* (GCACGTCCACACCTCCGATAA, TGGACCTTCATGTGCTTCCGCAG); *DUSP6* (TGGAACGAGAATACGGGCG, CTTACTGAAGCCACCTTCCA); *EZH1* (CGGCTCGGGATGGAGGATTAC, GCCTTTGCACCCATATTTGCC); *EZH2* (GCTTCCTACATCGTAAGTGCAA, GCTCCCTCCAAATGCTGGTA); *KDM6A* (TGAATCCTGCAACCAGCCTC, AGGTTACACAACTGAGCCTGTA); *KDM6B* (CACCCACTGTGGTCTGTTGT, CGCCTCAGTAACAGCCAGAT); *GAPDH* (AATGAAGGGGTCATTGATGG, AAGGTGAAGGTCGGAGTCAA). Human-specific mitochondrial element (CGGGAGCTCTCCATGCATTT, GACAGATACTGCGACATAGGGT); Human-mouse conserved mitochondrial element (GCTAAGACCCAAACTGGGATT, GGTTTGCTGAAGATGGCGGTA).

Next generation sequencing and data analysis

Total RNA of human ESCs and ffEPSCs were prepared in duplicate with the HiPure Total RNA Mini Kit (Magen). Samples were sequenced on Illumina HiSeq X Ten PE150 at Annoroad Gene Technology Co. Ltd.

Sequencing reads were aligned to the human genome build hg38/GRCh38 with the HISAT2 (Pertea et al., 2016). Raw counts were performed with FeatureCounts (Liao et al., 2013) using GENCODE v29 human gene annotation (Harrow et al., 2012). Raw counts were normalized for total read counts using the size factors computed by the Bioconductor package DESeq2 (Love et al., 2014). Differential expression analysis was performed using the default settings of DESeq2 with p-value of <0.05 and filtering out

genes with TPM less than 1. To generate the heatmap for differential gene expression, TPM values were scaled relative to the mean expression of each gene across all samples in R (http://www.r-project.org/). The Gene Expression Omnibus (GEO) accession number for the RNA-seq raw data reported in this work is GSE137208 and expression values were shown as TPM in Table S1. Functional annotation of significantly different transcripts and enrichment analysis were performed with Clusterprofiler (Yu et al., 2012).

To quantify the specific genes for zygotic genome activation stage, two RNA-seq datasets of human early embryo development (GSE44183 (Xue et al., 2013) and GSE36552 (Yan et al., 2013)) were aligned to the human genome with the HISAT2 aligner, and raw counts were normalized to TPM and filtering out genes with TPM less than 20. The ZGA-specificity score of each transcript was defined as follows: Score = meanA - (meanOther+2*sdOther). Where meanA is the mean expression of the samples in certain stage, and meanOther and sdOther are the mean and SD of the expression levels in the other samples, respectively. Therefore, a positive score indicated that the gene was expressed in a certain stage at a considerably higher level than in the rest of the stages. A gene with a score of >0.5 was considered as specifically expressed in a certain stage.

For Gene Set Enrichment Analysis (GSEA) we used normalized counts by DEseq2 as input. The ZGA specific genes were generated by the overlap of two RNA-seq datasets (GSE44183 and GSE36552). The H3K27me3 ChIP-seq data was downloaded from GEO database (GSE89303) and accordingly analyzed (Yang et al., 2017).

Seahorse cellular flux assays

XF24 Cell Culture Microplates were pre-coated with Matrigel 2 hours before cell seeding. Human ESCs and EPSCs were seeded onto Matrigel-coated plate and cultured for 6 hours. Then the culture media was changed by base media (unbuffered DMEM supplemented with 2 mM Glutamine, pH 7.3-7.4) 500 μl per well about 1 hour before

the assay. Selective chemical inhibitors with proper concentrations were added during the measurements. Cell glycolysis stress test were measured using an XF24 Extracellular Flux Analyzer. All the data were normalized to DNA concentration calibrated by CyQuant™ Cell Proliferation Assay kit (Thermofisher).

REFERENCES:

Harrow, J., Frankish, A., Gonzalez, J.M., Tapanari, E., Diekhans, M., Kokocinski, F., Aken, B.L., Barrell, D., Zadissa, A., Searle, S*., et al.* (2012). GENCODE: the reference human genome annotation for The ENCODE Project. Genome research *22*, 1760-1774.

Liao, Y., Smyth, G.K., and Shi, W. (2013). featureCounts: an efficient general purpose program for assigning sequence reads to genomic features. Bioinformatics *30*, 923-930.

Love, M.I., Huber, W., and Anders, S. (2014). Moderated estimation of fold change and dispersion for RNA-seq data with DESeq2. Genome Biology *15*, 550.

Pertea, M., Kim, D., Pertea, G.M., Leek, J.T., and Salzberg, S.L. (2016). Transcript-level expression analysis of RNA-seq experiments with HISAT, StringTie and Ballgown. Nature Protocols *11*, 1650-1667.

Xue, Z., Huang, K., Cai, C., Cai, L., Jiang, C.-y., Feng, Y., Liu, Z., Zeng, Q., Cheng, L., Sun, Y.E*., et al.* (2013). Genetic programs in human and mouse early embryos revealed by single-cell RNA sequencing. Nature *500*, 593-597.

Yan, L., Yang, M., Guo, H., Yang, L., Wu, J., Li, R., Liu, P., Lian, Y., Zheng, X., Yan, J*., et al.* (2013). Single-cell RNA-Seq profiling of human preimplantation embryos and embryonic stem cells. Nature Structural & Molecular Biology *20*, 1131-1139.

Yang, Y., Liu, B., Xu, J., Wang, J., Wu, J., Shi, C., Xu, Y., Dong, J., Wang, C., Lai, W*., et al.* (2017). Derivation of Pluripotent Stem Cells with In Vivo Embryonic and Extraembryonic Potency. Cell *169*, 243-257.e225.

Yu, G., Wang, L.-G., Han, Y., and He, Q.-Y. (2012). clusterProfiler: an R Package for Comparing Biological Themes Among Gene Clusters. OMICS: A Journal of Integrative Biology *16*, 284-287.
