# Supplementary material for: Derivation of feeder-free human extended pluripotent stem cells
Source: Stem Cell Reports. 2021 Jul 1;16(7):1686–96. doi: 10.1016/j.stemcr.2021.06.001 (PMC8282469; doi:10.1016/j.stemcr.2021.06.001)
Supplement: Document S2. Article plus supplemental information [file mmc3.pdf]

Stem Cell Reports
Report
OPEN ACCESS

Derivation of feeder-free human extended pluripotent stem cells

Ran Zheng,[1,6] Ting Geng,[1,6] Dan-Ya Wu,[2,6] Tianzhe Zhang,[1] Hai-Nan He,[2] Hai-Ning Du,[3] Donghui Zhang,[4,*] Yi-Liang Miao,[2,*] and Wei Jiang[1,5,*]

[1]Department of Biological Repositories, Frontier Science Center for Immunology and Metabolism, Medical Research Institute, Zhongnan Hospital of Wuhan University, Wuhan University, Wuhan 430071, China
[2]Institute of Stem Cell and Regenerative Biology, College of Animal Science and Veterinary Medicine, Huazhong Agricultural University, Wuhan 430070, China
[3]Hubei Key Laboratory of Cell Homeostasis, College of Life Sciences, Wuhan University, Wuhan 430071, China
[4]State Key Laboratory of Biocatalysis and Enzyme Engineering, National & Local Joint Engineering Research Center of High-throughput Drug Screening Technology, School of Life Science, Hubei University, Wuhan 430062, China
[5]Human Genetics Resource Preservation Center of Wuhan University, Wuhan 430071, China
[6]These authors contributed equally
*Correspondence: dongh.zhang@hubu.edu.cn (D.Z.), miaoyl@mail.hzau.edu.cn (Y.-L.M.), jiangw.mri@whu.edu.cn (W.J.)
https://doi.org/10.1016/j.stemcr.2021.06.001

SUMMARY

Human extended pluripotent stem cells (EPSCs), with bidirectional chimeric ability to contribute to both embryonic and extraembryonic lineages, can be obtained and maintained by converting conventional pluripotent stem cells using chemicals. However, the transition system is based on inactivated mouse fibroblasts, and the underlying mechanism is not clear. Here we report a Matrigel-based feeder-free method to convert human embryonic stem cells and induced pluripotent stem cells into EPSCs and demonstrate the extended pluripotency in terms of molecular features, chimeric ability, and transcriptome. We further identify chemicals targeting glycolysis and histone methyltransferase to facilitate the conversion to and maintenance of feeder-free EPSCs. Altogether, our data not only establish a feeder-free system to generate human EPSCs, which should facilitate the mechanistic studies of extended pluripotency and further applications, but also provide additional insights into the transitions among different pluripotent states.

INTRODUCTION

There are two distinct types of cells during mammalian early embryonic development: totipotent cells that harbor superior development potential and are able to give rise to the whole conceptus, including embryonic and extraembryonic tissues, and pluripotent cells that can contribute only to embryonic lineages composed of most of the organs. The blastomere before the morula stage is considered totipotent and gradually loses totipotency and converts to pluripotency during the first lineage specification of the embryo (Suwińska et al., 2008). Naive and primed pluripotency, two different metastable pluripotent states, have been deciphered in detail *in vivo*, or their counterparts have been, *in vitro*. Scientists have put much effort into the conversion of primed human embryonic stem cells (ESCs) into a naive state (Guo et al., 2016; Takashima et al., 2014; Theunissen et al., 2014), but not into a totipotent state yet. Whether totipotent cells could be stably maintained *in vitro* was thus a long-standing question and poorly studied, particularly for human totipotent-like cells (Lu and Zhang, 2015). Macfarlan and colleagues in 2012 first reported a transient two-cell-like state existing in mouse ESC cultures, which exhibited a transcriptome profile similar to that of an *in vivo* two-cell embryo and superior developmental potency to contribute to both embryonic and extraembryonic tissues (Macfarlan et al., 2012). However, after the culture of these sorted two-cell-like cells, they spontaneously turned into an ESC mix containing only less than 5% two-cell-like cells. In 2017, Yang and colleagues identified a cocktail medium called LCDM, which could culture mouse and human conventional ESCs and induced pluripotent stem cells (iPSCs) into a different pluripotent state, named "extended pluripotency" (Yang et al., 2017b). The extended pluripotent stem cells (EPSCs) exhibited outstanding developmental potential and bidirectional chimeric ability, contributing to both embryonic tissues and extraembryonic tissues, including yolk sac and placenta. Another group also independently established a chemical cocktail to maintain a mouse single eight-cell-stage blastomere *in vitro* as "expanded potential" stem cells (Yang et al., 2017a) and later succeeded in pig and human species (Gao et al., 2019). These two pioneering works gave rise to irreplaceable cell types compared with naive or primed pluripotent stem cells in terms of both developmental potential and research significance. However, both of the conversion conditions were based on feeder cells, which brought uncertain factors that could interfere with further molecular dissection and potential clinical application (Chen et al., 2014).

Here, we report an optimized feeder-free culture condition to convert conventional human ESCs and iPSCs to EPSCs. We characterize the transcriptome profiles during the conversion and find that the feeder-free EPSCs (ffEPSCs) exhibit more similarities to naive rather than primed ESCs, but are still different from the naive state.

 © 2021 The Author(s).
This is an open access article under the CC BY license (http://creativecommons.org/licenses/by/4.0/).

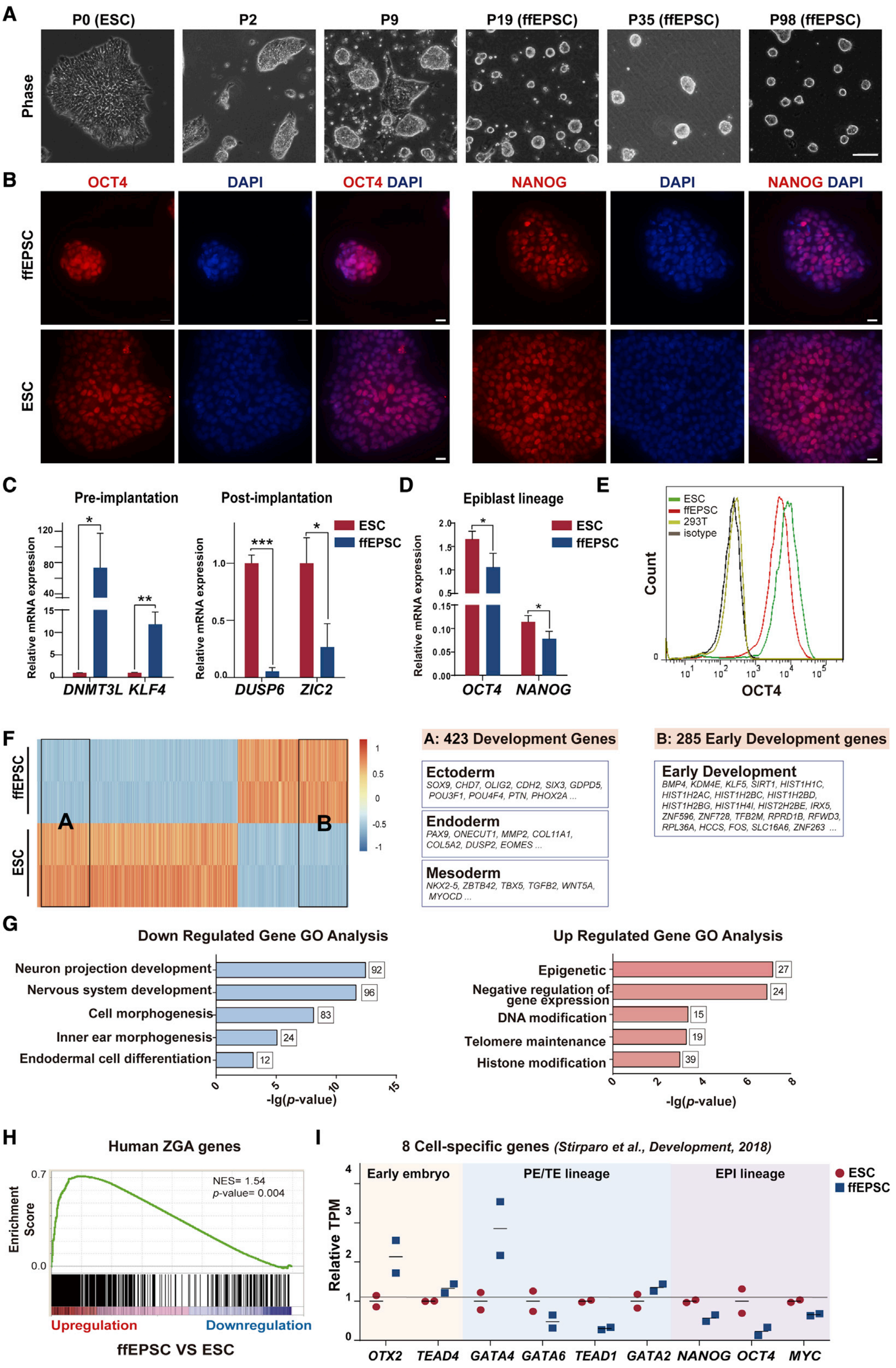

(legend on next page)

Human ffEPSCs express positive, but relatively lower, levels of core pluripotent genes, including OCT4 and NANOG, and highly express genes enriched in zygotic genome activation. More importantly, human EPSCs exhibit superior chimeric ability that contributes to both embryonic and extraembryonic lineages. We further explore the epigenetic and metabolic characters and accordingly improve the protocol by applying chemicals targeting glycolysis and histone methyltransferase.

RESULTS

Generation of human EPSCs under feeder-free conditions

Because the previous conditions under which human EPSCs were established, based on inactivated mouse embryonic fibroblasts, would hinder further mechanistic studies and potential clinical applications (Chen et al., 2014), we attempted to convert human ESCs into EPSCs without the feeder. After testing a panel of chemicals targeting pluripotency and differentiation, we eventually found that LCDM plus another two chemicals, IWR-1-endo and Y27632 (called LCDM-IY), with a high concentration of Matrigel, was able to convert human ESCs into dome-shaped EPSCs. When human ESCs were transferred to LCDM-IY medium, extensive cells flattened out and were differentiated, but domed colonies that were distinct from flat conventional ESC colonies emerged from passage 2 (Figure 1A). We handpicked those dome-shaped colonies for several passages until domed colonies steadily appeared around passage 20. Then, the ffEPSCs were maintained as domed colonies for more than 100 passages by single-cell dissociation every 2–3 days, followed by reseeding at a low split ratio of 1:10 (Figure 1A).

To characterize the pluripotency of converted ffEPSCs, we first checked the expression levels of the key pluripotent markers OCT4 and NANOG, and the ffEPSCs indeed maintained positive expression of OCT4 and NANOG proteins (Figure 1B). Since conventional human ESCs are considered primed pluripotency rather than naive pluripotency, we further confirmed the expression levels of pre-implantation and post-implantation genes as naive and primed markers, respectively (Geng et al., 2019; Guo et al., 2016; Takashima et al., 2014; Theunissen et al., 2014). By qRT-PCR analysis, we found that ffEPSCs did express significantly higher pre-implantation genes such as *DNMT3L* and *KLF4* but did not express or rarely expressed post-implantation markers, including *DUSP6* and *ZIC2* (Figure 1C). These data support the notion that ffEPSCs are much closer to an earlier naive pluripotent state than a later primed state.

To determine the robustness of the feeder-free method, we applied our conversion system to multiple pluripotent stem cell lines. The results of another human ESC line, H9, and one human iPSC line, PGP1 (Wang et al., 2014), demonstrated that the LCDM-IY feeder-free system supported generation of human ffEPSCs from various pluripotent stem cell lines. Similarly, many cells died or differentiated when transferred to the LCDM-IY system, but a few cells survived and formed dome-shaped colonies (Figures S1A and S1D). These ffEPSCs could be stably maintained from around passage 16 and positively expressed OCT4 and NANOG (Figures S1B and S1E). Further RNA analysis suggested that these ffEPSCs derived from different pluripotent stem cells highly expressed naive markers rather than primed markers (Figures S1C and S1F).

Very interestingly, we also found that the epiblast lineage genes *OCT4* and *NANOG* were downregulated in ffEPSCs by qRT-PCR analysis (Figure 1D). Since the EPSCs maintained positive expression of the core pluripotent genes OCT4 and NANOG (Figure 1B), we further performed flow cytometry analysis to quantitatively compare the protein levels. The result showed that the protein level of OCT4 was decreased compared with that in conventional ESCs (Figure 1E). To further investigate the molecular characters of ffEPSCs, we performed RNA-sequencing (RNA-seq) analysis for ffEPSCs and the parental ESCs. Compared with ESCs, the

Figure 1. Generation of human ffEPSCs from ESCs under feeder-free conditions

(A) The morphology of cells during the transition of human ESCs into ffEPSCs. Scale bar, 100 μm.

(B) Human ffEPSCs showed positive staining of NANOG and OCT4. ESCs served as positive control. Scale bars, 20 μm.

(C) Expression patterns of pre-implantation genes and post-implantation genes in ffEPSCs compared with ESCs (three independent experiments). $^{*}p < 0.05$, $^{***}p < 0.001$.

(D) RNA expression levels of *OCT4* and *NANOG* in ffEPSCs and ESCs analyzed by qRT-PCR (three independent experiments). $^{*}p < 0.05$.

(E) Human ffEPSCs showed lower signal for OCT4 compared with ESCs by flow cytometric analysis (three independent experiments).

(F) Differentially expressed transcripts between ffEPSCs and ESCs revealed by RNA-seq and developmental genes listed as different categories.

(G) Gene ontology (GO) analysis of the up- and downregulated genes in ffEPSCs.

(H) GSEA showed the expression pattern of human zygotic genome activation (ZGA) genes in human ffEPSCs and ESCs.

(I) Expression patterns of representative early embryo-specific genes and primitive endoderm and trophectoderm (PE/TE) genes in ffEPSCs and ESCs. EPI, epiblast.

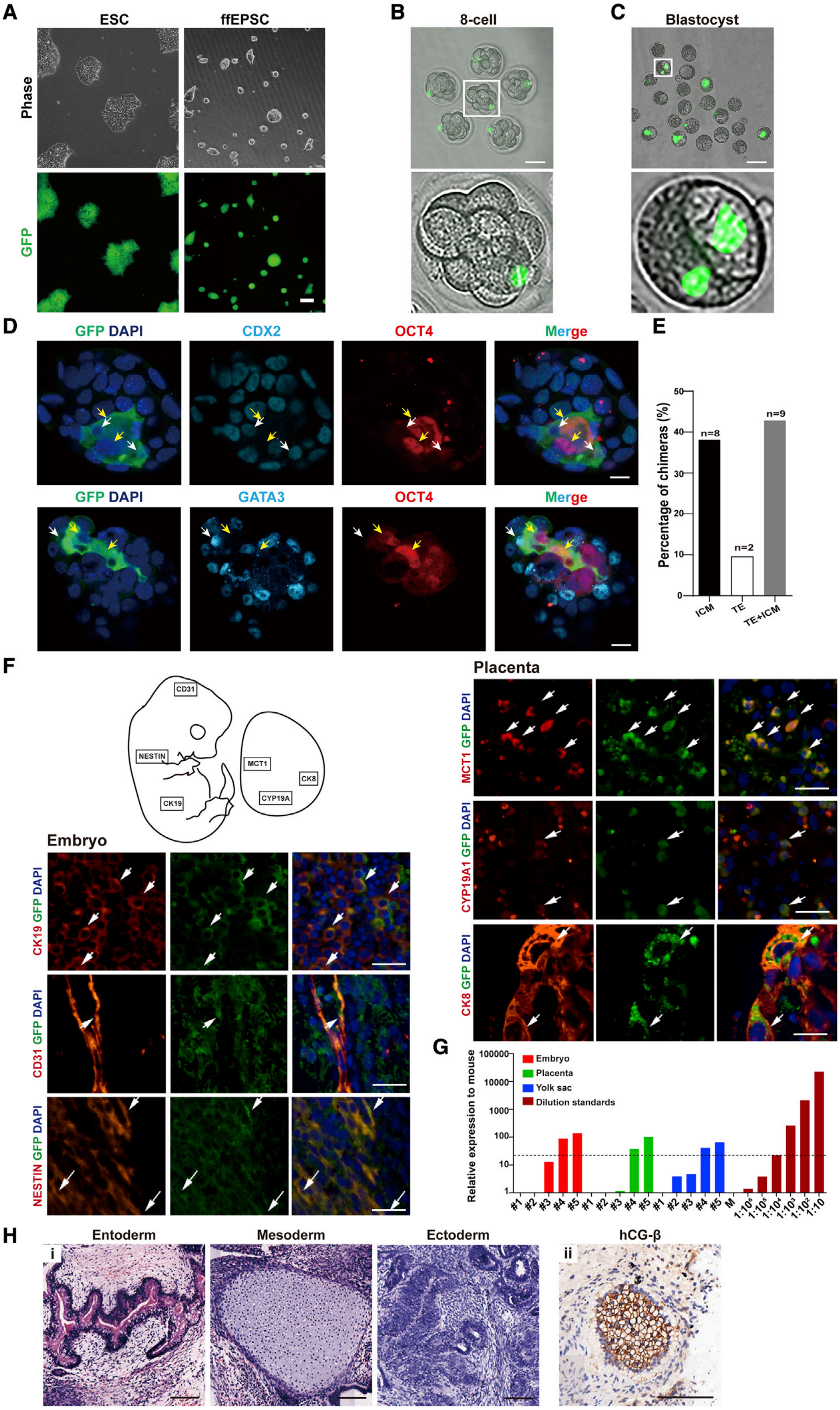

(legend on next page)

genes downregulated in ffEPSCs were significantly enriched in developmental genes and cell differentiation-related genes. By looking into the different categories of developmental genes, we found that ffEPSCs expressed higher levels of early development genes and lower levels of embryonic developmental genes, including ectodermal, endodermal, and mesodermal genes (Figure 1F). In addition, the upregulated genes in ffEPSCs were noteworthily associated with epigenetics, DNA modification, and histone modification (Figures 1F and 1G). Since the lower expression pattern of germ-layer genes is a characteristic of naive pluripotency compared with primed pluripotency, we further performed gene set enrichment analysis (GSEA) and, more interestingly, human zygotic genome activation genes were significantly upregulated in ffEPSCs (Figure 1H), and indeed, naive-specific genes were significantly upregulated, but primed-specific genes were significantly downregulated in EPSCs compared with ESCs (Figure S1G).

Furthermore, we found that early embryo-specific genes such as *OTX2* (Wu et al., 2018) and *TEAD4* (Xia et al., 2019) were highly expressed in ffEPSCs, while primitive endoderm and trophectoderm (TE) lineage genes (*GATA4* and *GATA6*, *TEAD1* and *GATA2*, respectively) (Li et al., 2018) were rarely expressed or downregulated (Figure 1I), suggesting an earlier stage of ffEPSCs than the naive state. Consistent with this notion, we also found that the epiblast lineage genes (*OCT4*, *NANOG*, and *MYC*) were downregulated in ffEPSCs (Figure 1I) and qRT-PCR analysis confirmed the decreased expression levels of *OCT4* and *NANOG* in ffEPSCs (Figure 1D). In addition, recent comprehensive data mining identified ten genes as specific markers of the eight-cell stage based on a number of RNA-seq datasets (Stirparo et al., 2018). We checked our data, and indeed, nine of those ten eight-cell-specific genes showed higher expression in ffEPSCs compared with parental ESCs (*KMT5A* highly expressed in both ESC and ffEPSC samples) (Figure 1I). Moreover, the higher expression levels of human zygotic activation genes and lower levels of *OCT4* and *NANOG* suggested that ffEPSCs might represent an earlier pluripotent state different from primed or naive ESCs, as early embryos of humans and primates did not express OCT4 or NANOG until the blastocyst stage (Li et al., 2018). These data together support the conclusion that ffEPSCs exhibit a transcription pattern closer to that of human early embryos.

ffEPSCs exhibited bidirectional chimeric ability

Next, we examined the bidirectional chimeric ability of the ffEPSCs. We first constructed a human ESC line with a stably expressed GFP reporter under the control of the EF1a promoter, and then converted this reporter line to dome-shaped ffEPSCs (Figure 2A). We microinjected a single GFP-labeled ffEPSC into eight-cell-stage mouse embryos (Figure 2B) followed by 36–48 h culture *in vitro*, and GFP signals were observed in embryos entering the blastocyst stage (Figure 2C; 16 of 62 checked embryos), suggesting ffEPSCs could support early embryo development. Furthermore, we performed immunostaining for the chimeric blastocysts to check the identity of GFP-positive cells. The result showed that GFP co-stained with the inner cell mass marker OCT4 or the TE marker CDX2 or GATA3 (Home et al., 2009) (Figure 2D) within the same blastocyst. Among the 19 GFP-positive blastocysts we checked (from two independent batches of blastocyst injections), 9 showed co-staining of both GFP^+/OCT^+ and GFP^+/TE^+, 8 showed co-staining of GFP^+/OCT^+ only, and 2 showed co-staining of GFP^+/TE^+ only (Figure 2E).

We further determined the fate of GFP-positive cells in E13.5 chimeric embryos and placenta. The immune-fluorescence assay of GFP and lineage markers including NESTIN (ectoderm), CK19 (endoderm), and CD31 (mesoderm) showed that ffEPSCs contributed to three germ-layer lineages; meanwhile, the GFP signal was observed to co-localize with the placenta markers CK8, MCT1 (Posfai

Figure 2. Human ffEPSCs exhibit bidirectional chimeric ability
(A) Morphology and GFP fluorescence signal of ESCs and ffEPSCs. Scale bar, 100 μm.
(B) Mouse eight-cell embryos injected with single GFP-labeled ffEPSC. Scale bar, 50 μm.
(C) The mouse-human chimeric embryos developed to blastocyst stage. Scale bar, 100 μm.
(D) GFP-labeled ffEPSCs contributed to both inner cell mass (ICM, marked with OCT4) and trophectoderm lineage (TE, marked with CDX2 or GATA3) in mouse embryos. The arrows indicate the co-stained cells. Scale bars, 10 μm.
(E) Statistical results of bidirectional chimeric assay in (D) ("n" stands for number of independent mouse embryos).
(F) Immunofluorescent staining of GFP (green) and lineage markers (red) in a section of an E13.5 embryo injected with GFP-labeled ffEPSCs. NESTIN represents ectoderm-derived neural tissue, CK19 represents endoderm-derived gland tissue, CD31 represents mesoderm-derived endothelial cells; and CK8 represents pan-placental markers, MCT1 represents syncytiotrophoblast I, and CYP19A1 represents estrogen synthesis cells. Scale bars, 25 μm.
(G) Quantitative PCR measurements of human mitochondrial DNA indicate the presence of human cells in mouse embryos, placentas, and yolk sac at E13.5 following injection of a single ffEPSC at the eight-cell stage; a series of human-mouse cell dilutions was run in parallel to estimate the degree of human cell contribution. Black dotted line highlights level equivalent to 1:100,000 diluted standard.
(H) (i) Hematoxylin-eosin staining (for three germ layers) in the teratoma section and (ii) immunofluorescent staining of hCG-β (for extraembryonic lineage). Scale bars, 100 μm.

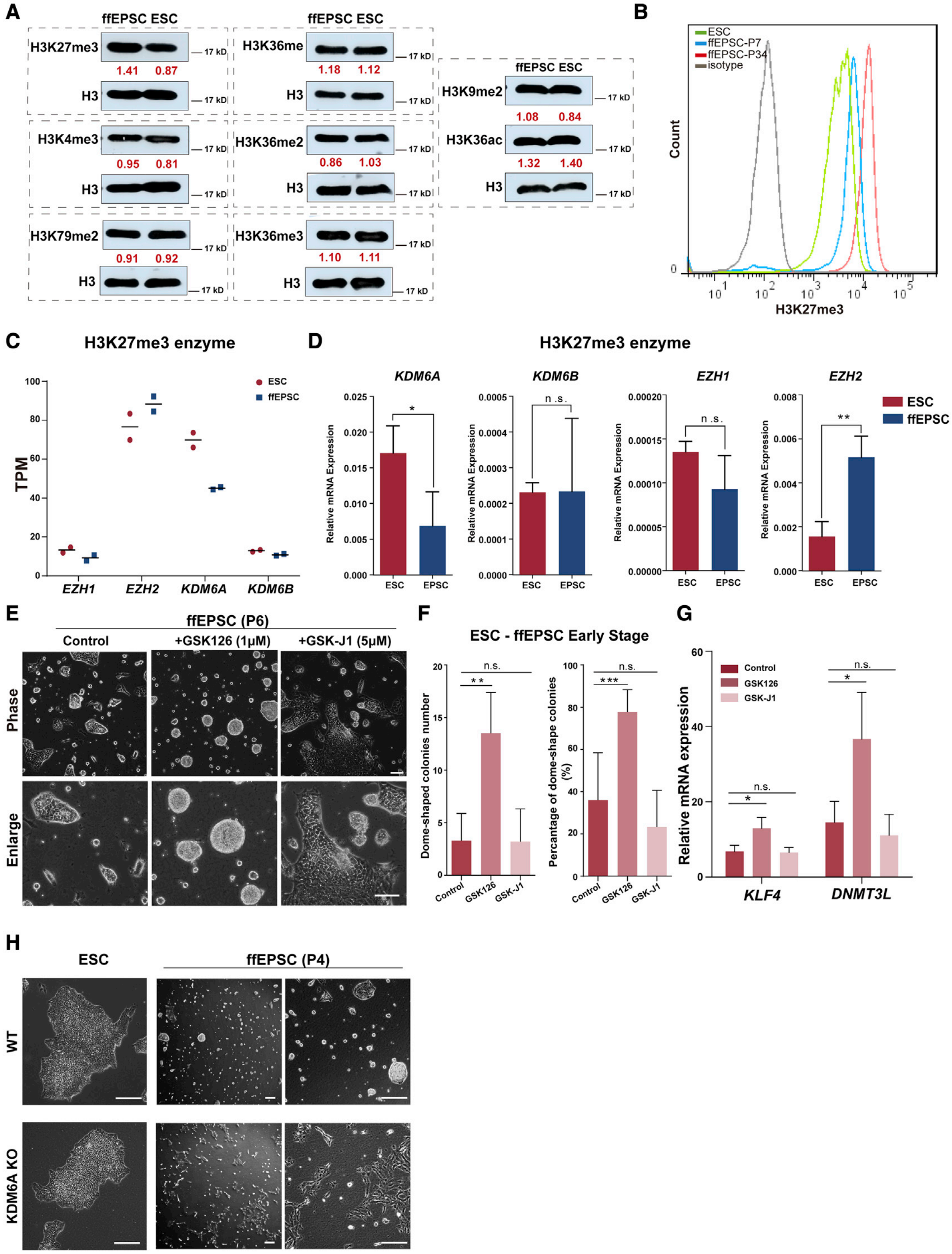

Figure 3. Dynamic H3K27me3 during the acquisition of extended pluripotency

(A) The pattern of histone modifications H3K27me3 and H3K4me3, H3K9me2, H3K36me, H3K36me2, H3K36me3, H3K79me2, and H3K36ac in human ESCs and ffEPSCs analyzed by western blotting. Total H3 served as the loading control.

(B) Human ffEPSCs showed higher H3K27me3 signal compared with ESCs by flow cytometric analysis.

(C) Expression of H3K27me3 enzyme in ESCs and ffEPSCs revealed by RNA-seq.

(D) qRT-PCR analysis measured the expression levels of H3K27me3-related enzymes in ESCs and ffEPSCs (three independent experiments). $^*p < 0.05$, $^{**}p < 0.01$.

(legend continued on next page)

et al., 2021), and CYP19A1 (encoding aromatase for estrogen synthesis) (Tsang et al., 2017) (Figure 2F), indicating that ffEPSCs could contribute to various placental cell lineages. We also collected a series of embryos for DNA analysis using primers specific for human mitochondrial DNA and for mouse-human conserved DNA sequence, and the result indicated that ffEPSCs indeed contributed to both embryonic and extraembryonic (placenta and yolk sac) lineages to a certain extent at the E13.5 stage (Figure 2G). The observation that three well-differentiated germ layer lineages and an hCG-β-positive extraembryonic lineage were detected in the ffEPSC-derived teratoma also supports this notion (Figure 2H). Taken together, the chimeric embryo analysis and the teratoma assay showed that the converted ffEPSCs exhibited bidirectional chimeric ability and could differentiate into both embryonic and extraembryonic lineages *in vivo*, indicating that ffEPSCs are more likely to have totipotency-like characteristics than conventional ESCs.

GSK126 promoted the transition from ESCs to ffEPSCs

The top gene ontology terms of the upregulated genes in ffEPSCs compared with ESCs were for enriched epigenetic regulation, including the term of "histone modifications" (Figure 1G), which drove us to ask whether epigenetic modifications might facilitate the transition. In fact, epigenetic modifications played a vital role in pluripotency transition (Geng et al., 2019). We first comprehensively analyzed the global levels of major histone modifications. The western blotting results showed that the H3K27me3 level was specifically higher in EPSCs (Figure 3A), although H3K4me3, H3K79me2, H3K36ac, and other modifications between these two cell types were comparable (Figure 3A). To further confirm the differential global H3K27me3 levels, we performed flow cytometric analysis. Fully converted ffEPSCs (passage 34) exhibited significantly stronger signal than ESCs, while the intermediate (ffEPSCs at passage 7) showed a mild global H3K27me3 signal (Figure 3B). We further surveyed the RNA levels of methyltransferases and demethylases responsive to H3K27me3 modification. RNA-seq data suggested that *EZH2*, encoding the H3K27 methyltransferase, was higher and *KDM6A*, encoding the H3K27 demethylase, was lower in EPSCs than in ESCs, while *EZH1* and *KDM6B* were less expressed (Figure 3C). The qRT-PCR assay also validated the expression pattern (Figure 3D), which was consistent with the higher global H3K27me3 level in EPSCs.

Next, we investigated the effects of GSK126 targeting the H3K27 methyltransferase EZH2 (McCabe et al., 2012) and GSK-J1 targeting the H3K27 demethylases (Kruidenier et al., 2012) during the EPSC transition. Interestingly, we observed more domed colonies in the GSK126-treated group, but not in the GSK-J1 group (Figures 3E and 3F). Gene expression analysis also supported this notion that the EZH2 inhibitor GSK126 facilitated EPSC-related genes expression (Figure 3G). Similar results were observed for H9-derived ffEPSCs (Figures S2A and S2B) and iPSC PGP1-derived ffEPSCs (Figure S2C). In addition, we observed that KDM6A-knockout ESCs failed to be converted to ffEPSCs (Figure 3H), which, together with the effect of EZH2 inhibitor GSK126, strongly supported that histone modification plays an important role in acquiring human extended pluripotency from primed pluripotency.

To better understand why EPSCs have a high level of H3K27me3 but the EZH2 inhibitors facilitate the conversion of EPSCs, we further analyzed the genome-wide distribution of H3K27me3. In embryonic development, major histone methylations happen in coding genes, regulator genes such as ncRNA, and repeat elements (LTR, LINE, SINE), but coding genes and regulator genes are believed to be more conclusive for cell fate regulation. We surveyed the H3K27me3 chromatin immunoprecipitation-sequencing (ChIP-seq) data (GSE89301) and the result showed that coding genes and ncRNA exhibited lower H3K27me3 levels in EPSCs, and higher global H3K27me3 was mainly contributed by the repeat elements (Figure S2D). This finding indicates that the global H3K27me3 level did not represent the transcriptional regulation of coding genes, whereas H3K27me3 might actually serve as a negative regulator in acquiring human extended pluripotency. Nonetheless, further experiments would absolutely be needed to understand how H3K27 methylation regulates the transition.

Chemicals targeting glycolysis facilitated the maintenance of ffEPSCs

Since a significant portion of the glycolysis-associated genes in ffEPSCs showed downregulation compared with ESCs (10/47, Figure 4A), but very few tricarboxylic acid cycle-associated genes changed (Figure 4B), we wondered whether glycolysis plays a special role in ffEPSC maintenance. We treated ffEPSCs with glycolysis inhibitors, including 2-deoxy-D-glucose (2-DG) and aurintricarboxylic acid (ATA) (McCune et al., 1989). The results showed that either 2-DG or ATA treatment group increased the percentage of domed colonies, although the numbers of colonies

(E) Morphology of ffEPSCs cultured with chemical inhibitors GSK-126 and GSK-J1. Scale bars, 100 μm.
(F) The number and percentage of dome-shaped colonies of ffEPSCs cultured with GSK-126 or GSK-J1 (three independent experiments). $^{**}p < 0.01$, $^{***}p < 0.001$.
(G) Gene expression levels of ffEPSCs cultured with GSK-126, GSK-J1, or vehicle control (three independent experiments). $^{*}p < 0.05$.
(H) KDM6A-KO ESCs failed to convert to ffEPSCs. Scale bars, 100 μm. KO, knockout; WT, wild type.

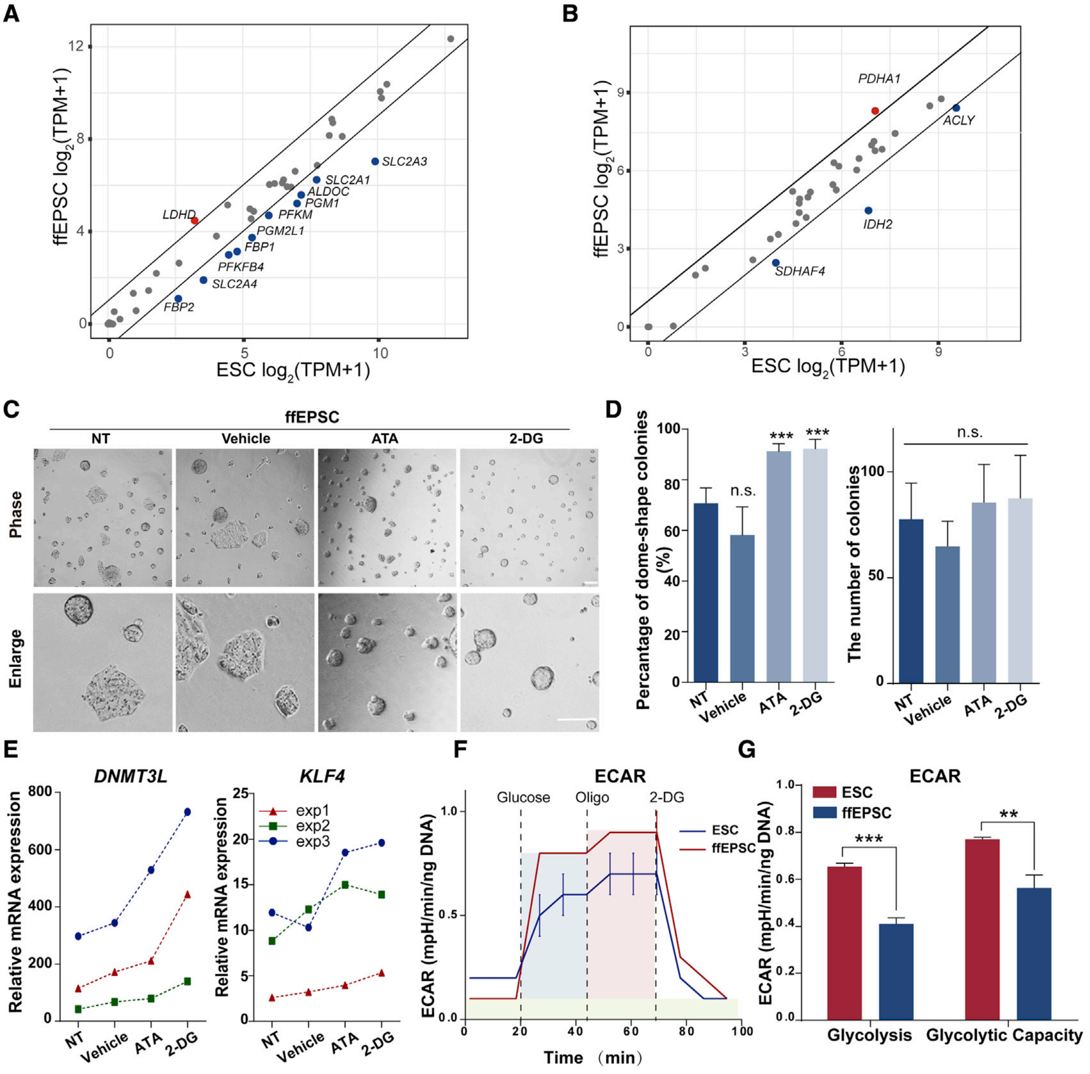

Figure 4. Chemicals facilitated the generation and maintenance of human EPSCs

(A) Plot of the expression levels of glycolysis-related genes in ESCs and ffEPSCs. The upregulated genes are colored in red (1/47) and downregulated genes in blue (10/47).

(B) The plot of expression levels of TCA-related genes in ESCs and ffEPSCs. The up-regulated genes are colored in red (1/34) and blue for downregulated gene (3/34).

(C) Morphology of ffEPSCs cultured with the chemical inhibitors 2-DG and ATA. NT, non-treated. Scale bars, 100 μm.

(D) The number and percentage of dome-shaped colonies of ffEPSCs cultured with 2-DG or ATA (five independent experiments). ***$p < 0.001$.

(E) qRT-PCR analysis of pre-implantation genes after treatment with glycolytic inhibitors (three independent experiments).

(F and G) ECAR analysis of (F) glycolytic rate and (G) capacity of ffEPSCs measured by Seahorse flux compared with ESCs (three independent experiments). **$p < 0.01$, ***$p < 0.001$.

were similar (Figures 4C and 4D). Consistent with the result, the ffEPSCs treated with glycolysis inhibitors indeed expressed higher levels of earlier stage genes, such as *DNMT3L* and *KLF4* (Figure 4E). In addition, we performed the Seahorse flux analysis and the result showed that the extracellular acidification rate (ECAR) of EPSCs was significantly decreased in EPSCs (Figures 4F and 4G), indicating lower glycolytic activity in ffEPSCs compared with ESCs, which was consistent with the report that naive mouse ESCs displayed lower glycolysis levels than primed epiblast stem cells (EpiSCs) and human ESCs (Zhou et al., 2012). To further confirm the effects of glycolysis inhibitors, we chose iPSC- or H9-derived ffEPSCs for a similar assay. 2-DG and ATA indeed increased the percentage of domed colonies,

yet with little effect on the number of colonies (Figures S3A, S3B, S3D, and S3E). Supporting this notion, 2-DG- or ATA-treated iPSC-derived ffEPSCs expressed higher *DNMT3L* and *KLF4* (Figures S3C and S3F). These data together indicated that the application of glycolysis inhibitors improved the maintenance of ffEPSCs.

DISCUSSION

In this study, we started with optimizing the feeder-free transition and maintenance conditions for human EPSCs and then characterized the molecular and biological properties of the converted ffEPSCs in detail. Our data demonstrated that human ffEPSCs exhibited a transcriptome similar to that of earlier embryos, including relatively lower OCT4/NANOG levels and an upregulated zygotic activation gene set. Moreover, we further reported that a chemical inhibitor targeting glycolysis or histone H3K27me3 methyltransferase EZH2 could greatly facilitate the generation and maintenance of ffEPSCs, albeit the underlying mechanisms are waiting for further investigation.

EPSC is a distinct cell category from known pluripotent cells, with unique and valuable characteristics. OCT4 and NANOG are functionally expressed in both naive and primed pluripotent ESCs and iPSCs, but are not or are less expressed in totipotent-like cells, including either early human embryos before the later morula stage (Li et al., 2018) or two-cell-like cells existing in mouse ESC cultures (Macfarlan et al., 2012). We observed that ffEPSCs expressed OCT4 and NANOG, but at a relatively lower level compared with human ESCs (Figures 1D and 1E). However, it remains unknown how OCT4 and NANOG are downregulated in ffEPSCs, or even in early development. This pattern, together with the transcriptome analysis (Figures 1F–1I and S1G), strongly indicates that ffEPSCs exhibit a state very distinct from, but earlier than, naive or primed pluripotency. In addition, EPSCs were recently shown to be able to greatly advance the efficiency of generating gene-targeting mouse models (Li et al., 2019), and contribute to interspecies chimerism (Tan et al., 2021), indicating their significance and broad application. Herein, we believe that ffEPSCs can work as a key node between totipotency and pluripotency, and can serve as an advanced cell model to study early development at the molecular level in the future. The feeder-free conditions to convert human pluripotent ESCs into EPSCs, established in this paper, should further promote the study of totipotency and application of EPSCs with superior chimeric ability.

Cellular metabolism is a complex and highly coordinated life-sustaining biochemical reaction that occurs within a cell that transforms or uses energy to maintain its survival. Several studies have analyzed the cellular metabolism patterns in naive and primed states. Naive mouse ESCs displayed lower glycolysis levels than primed EpiSCs and human ESCs (Zhou et al., 2012). Consistent with this, here we revealed that human ffEPSCs exhibited lower glycolytic activity than conventional human ESCs (Figure 4). Furthermore, a distinct metabolism pattern may actively participate in cell fate determination. For instance, the ratio of α-ketoglutarate to succinic acid was critical to naive and primed pluripotency: a high ratio of α-ketoglutarate to succinic acid promoted differentiation of human ESCs (TeSlaa et al., 2016). But in a mouse model, a high ratio of α-ketoglutarate to succinic acid promoted mouse ESC self-renewal (Carey et al., 2015). In human ffEPSCs, here we reported that treatment with glycolytic inhibitors was beneficial to the maintenance of extended pluripotency. The dependence on oxidative metabolism supports the resemblance of these ffEPSCs to earlier embryo stages, and our data based on chemical inhibitors demonstrate a distinct metabolism pattern that may actively participate in cell fate determination. In addition, epigenetics serves as a pre-transcriptional factor that has been implicated in vital roles in development. Generally, H3K27me3 serves as a repressive marker to alter the chromatin state in naive-state pluripotency. Importantly, the global H3K27me3 level shows dynamic remodeling in the early stage blastomere, which is maintained in the two- to eight-cell stages, decreases in the eight-cell to morula stages, and is reestablished in the inner cell mass (Zhang et al., 2009). Moreover, histone modification was reported to play a critical role in the transition of mouse cells primed to the naive state (Zhang et al., 2016). Our data that chemical inhibition of the methyltransferase EZH2 facilitated the conversion of ffEPSCs, and genetic depletion of the demethylase KDM6A blocked the conversion of ffEPSCs, suggest that the H3K27me3 level serves as a negative regulator in acquiring human extended pluripotency.

In summary, we established feeder-free conditions to convert human pluripotent ESCs into EPSCs with bidirectional chimeric ability. We further profiled histone modifications and glucose metabolic flux and provided proof-of-concept evidence that modulation of H3K27me3-related enzymes or glycolytic activity could facilitate the conversion and maintenance of human ffEPSCs. Our findings provide a metabolic and epigenetic insight into the acquisition of extended pluripotency.

EXPERIMENTAL PROCEDURES

Maintenance and conversion of human ESCs to ffEPSCs

The human ESC lines HUES8 and H9 and the iPSC line PGP1 were normally maintained on Matrigel (Corning)-coated (1:100) plates with mTeSR1 (STEMCELL Technologies) supplemented with 1% penicillin-streptomycin (Jiang et al., 2013). For the conversion of

ESCs to EPSCs, single cells treated with Accutase were moved to Matrigel-coated six-well plates with mTeSR1. On the next day, the medium was exchanged to the LCDM-IY medium. LCDM-IY medium was based on a mix of knockout DMEM/F12 and neurobasal medium (1:1), supplemented with 0.5× B27 supplement, 0.5× N2 supplement, 5% knockout serum replacement, 1% GlutaMAX, 1% non-essential amino acids, 1% penicillin-streptomycin, 0.1 mM β-mercaptoethanol, and an additional six inhibitors, comprising recombinant human LIF (10 ng/mL), CHIR99021 (1 μM), (S)-(+)-dimethindene maleate (2 μM), minocycline hydrochloride (2 μM), IWR-endo-1 (1 μM), and Y-27632 (2 μM). One or two days later, the cells were collected using trypsin and reseeded on Matrigel-coated plates (1:30) in LCDM-IY medium and cultured with daily change of the medium. Established ffEPSCs were passaged using TrypLE about every 3 days, while conventional HUES8 cells were passaged using Accutase every 5 days.

Statistical methods

All experiments were performed in at least three independent experiments. Data were presented as means ± SD. Statistical comparisons were conducted via Student's t test (two-tailed, equal variance), and p values were shown with $*p < 0.05$, $**p < 0.01$, $***p < 0.001$.

Accession numbers

The GEO accession numbers for the next-generation sequencing data reported in this work are GSE137208 (RNA-seq for ESCs/ffEPSCs), GSE44183 and GSE36552 (RNA-seq for human early embryo development samples), and GSE89303 (H3K27me3 ChIP-seq data).

SUPPLEMENTAL INFORMATION

Supplemental information can be found online at https://doi.org/10.1016/j.stemcr.2021.06.001.

AUTHOR CONTRIBUTIONS

W.J. and D.Z. conceived the project and designed the experiment together with R.Z. and T.G. T.G. and R.Z. performed most of the bench experiments and T.Z. analyzed the NGS data. D-Y.W. performed the chimera assay with help from H-N.H., and R.Z. and T.G. performed the embryo staining and image capture. H-N.D. provided experimental material regarding histone modification analysis. W.J. and Y-L.M. supervised the project. W.J., T.G., R.Z., D.Z., and Y-L.M. wrote the manuscript. All authors contributed to and approved the final manuscript.

CONFLICTS OF INTERESTS

R.Z., T.G., and W.J. have filed a patent application (202010062150.0) related to this work through Wuhan University.

ACKNOWLEDGMENTS

We would like to thank Jing Lv, Ran Liu, Liyan Wang, Chengli Dou, and other laboratory members for technical help and discussion. All mouse experimental protocols were approved by the Animal Management and Ethics Committee, Huazhong Agriculture University. W.J. was supported by grants from the National Key Research and Development Program of China (2016YFA0503100), the National Natural Science Foundation of China (31970608), the Science and Technology Department of Hubei Province Key Project (2018ACA159), the Medical Science Advancement Program of Wuhan University (TFJC2018005), and the Fundamental Research Funds for the Central Universities China (2042021kf0207). D.Z. was funded by the Ministry of Science and Technology of the People's Republic of China (National Science and Technology Major Project, grant 2018YFA0109100) and the National Natural Science Foundation of China (31871496).

Received: May 1, 2021
Revised: May 31, 2021
Accepted: June 1, 2021
Published: July 1, 2021

REFERENCES

Carey, B.W., Finley, L.W.S., Cross, J.R., Allis, C.D., and Thompson, C.B. (2015). Intracellular α-ketoglutarate maintains the pluripotency of embryonic stem cells. Nature *518*, 413–416.

Chen, K.G., Mallon, B.S., McKay, R.D.G., and Robey, P.G. (2014). Human pluripotent stem cell culture: considerations for maintenance, expansion, and therapeutics. Cell Stem Cell *14*, 13–26.

Gao, X., Nowak-Imialek, M., Chen, X., Chen, D., Herrmann, D., Ruan, D., Chen, A.C.H., Eckersley-Maslin, M.A., Ahmad, S., Lee, Y.L., et al. (2019). Establishment of porcine and human expanded potential stem cells. Nat. Cell Biol. *21*, 687–699.

Geng, T., Zhang, D., and Jiang, W. (2019). Epigenetic regulation of transition among different pluripotent states: concise review. Stem cells *37*, 1372–1380.

Guo, G., von Meyenn, F., Santos, F., Chen, Y., Reik, W., Bertone, P., Smith, A., and Nichols, J. (2016). Naive pluripotent stem cells derived directly from isolated cells of the human inner cell mass. Stem Cell Rep. *6*, 437–446.

Home, P., Ray, S., Dutta, D., Bronshteyn, I., Larson, M., and Paul, S. (2009). GATA3 is selectively expressed in the trophectoderm of peri-implantation embryo and directly regulates Cdx2 gene expression. J. Biol. Chem. *284*, 28729–28737.

Jiang, W., Wang, J., and Zhang, Y. (2013). Histone H3K27me3 demethylases KDM6A and KDM6B modulate definitive endoderm differentiation from human ESCs by regulating WNT signaling pathway. Cell Res. *23*, 122–130.

Kruidenier, L., Chung, C.W., Cheng, Z., Liddle, J., Che, K., Joberty, G., Bantscheff, M., Bountra, C., Bridges, A., Diallo, H., et al. (2012). A selective jumonji H3K27 demethylase inhibitor modulates the proinflammatory macrophage response. Nature *488*, 404–408.

Li, H., Zhao, C., Xu, J., Xu, Y., Cheng, C., Liu, Y., Wang, T., Du, Y., Xie, L., Zhao, J., et al. (2019). Rapid generation of gene-targeted EPS-derived mouse models through tetraploid complementation. Protein Cell *10*, 20–30.

Li, L., Guo, F., Gao, Y., Ren, Y., Yuan, P., Yan, L., Li, R., Lian, Y., Li, J., Hu, B., et al. (2018). Single-cell multi-omics sequencing of human early embryos. Nat. Cell Biol. *20*, 847–858.

Lu, F., and Zhang, Y. (2015). Cell totipotency: molecular features, induction, and maintenance. Natl. Sci. Rev. *2*, 217–225.

Macfarlan, T.S., Gifford, W.D., Driscoll, S., Lettieri, K., Rowe, H.M., Bonanomi, D., Firth, A., Singer, O., Trono, D., and Pfaff, S.L. (2012). Embryonic stem cell potency fluctuates with endogenous retrovirus activity. Nature *487*, 57–63.

McCabe, M.T., Ott, H.M., Ganji, G., Korenchuk, S., Thompson, C., Van Aller, G.S., Liu, Y., Graves, A.P., Della Pietra, A., 3rd, Diaz, E., et al. (2012). EZH2 inhibition as a therapeutic strategy for lymphoma with EZH2-activating mutations. Nature *492*, 108–112.

McCune, S.A., Foe, L.G., Kemp, R.G., and Jurin, R.R. (1989). Aurintricarboxylic acid is a potent inhibitor of phosphofructokinase. Biochem. J. *259*, 925–927.

Posfai, E., Schell, J.P., Janiszewski, A., Rovic, I., Murray, A., Bradshaw, B., Yamakawa, T., Pardon, T., El Bakkali, M., Talon, I., et al. (2021). Evaluating totipotency using criteria of increasing stringency. Nat. Cell Biol. *23*, 49–60.

Stirparo, G.G., Boroviak, T., Guo, G., Nichols, J., Smith, A., and Bertone, P. (2018). Integrated analysis of single-cell embryo data yields a unified transcriptome signature for the human pre-implantation epiblast. Development *145*, dev158501.

Suwińska, A., Czołowska, R., Ożdżeński, W., and Tarkowski, A.K. (2008). Blastomeres of the mouse embryo lose totipotency after the fifth cleavage division: expression of Cdx2 and Oct4 and developmental potential of inner and outer blastomeres of 16- and 32-cell embryos. Dev. Biol. *322*, 133–144.

Takashima, Y., Guo, G., Loos, R., Nichols, J., Ficz, G., Krueger, F., Oxley, D., Santos, F., Clarke, J., Mansfield, W., et al. (2014). Resetting transcription factor control circuitry toward ground-state pluripotency in human. Cell *158*, 1254–1269.

Tan, T., Wu, J., Si, C., Dai, S., Zhang, Y., Sun, N., Zhang, E., Shao, H., Si, W., Yang, P., et al. (2021). Chimeric contribution of human extended pluripotent stem cells to monkey embryos ex vivo. Cell *184*, 2020–2032.e2014.

TeSlaa, T., Chaikovsky, A.C., Lipchina, I., Escobar, S.L., Hochedlinger, K., Huang, J., Graeber, T.G., Braas, D., and Teitell, M.A. (2016). α-Ketoglutarate accelerates the initial differentiation of primed human pluripotent stem cells. Cell Metab. *24*, 485–493.

Theunissen, T.W., Powell, B.E., Wang, H., Mitalipova, M., Faddah, D.A., Reddy, J., Fan, Zi P., Maetzel, D., Ganz, K., Shi, L., et al. (2014). Systematic identification of culture conditions for induction and maintenance of naive human pluripotency. Cell Stem Cell *15*, 471–487.

Tsang, J.C.H., Vong, J.S.L., Ji, L., Poon, L.C.Y., Jiang, P.Y., Lui, K.O., Ni, Y.B., To, K.F., Cheng, Y.K.Y., Chiu, R.W.K., et al. (2017). Integrative single-cell and cell-free plasma RNA transcriptomics elucidates placental cellular dynamics. Proc. Natl. Acad. Sci. U S A *114*, E7786–E7795.

Wang, G., McCain, M.L., Yang, L., He, A., Pasqualini, F.S., Agarwal, A., Yuan, H., Jiang, D., Zhang, D., Zangi, L., et al. (2014). Modeling the mitochondrial cardiomyopathy of Barth syndrome with induced pluripotent stem cell and heart-on-chip technologies. Nat. Med. *20*, 616–623.

Wu, J., Xu, J., Liu, B., Yao, G., Wang, P., Lin, Z., Huang, B., Wang, X., Li, T., Shi, S., et al. (2018). Chromatin analysis in human early development reveals epigenetic transition during ZGA. Nature *557*, 256–260.

Xia, W., Xu, J., Yu, G., Yao, G., Xu, K., Ma, X., Zhang, N., Liu, B., Li, T., Lin, Z., et al. (2019). Resetting histone modifications during human parental-to-zygotic transition. Science *365*, 353.

Yang, J., Ryan, D.J., Wang, W., Tsang, J.C.-H., Lan, G., Masaki, H., Gao, X., Antunes, L., Yu, Y., Zhu, Z., et al. (2017a). Establishment of mouse expanded potential stem cells. Nature *550*, 393–397.

Yang, Y., Liu, B., Xu, J., Wang, J., Wu, J., Shi, C., Xu, Y., Dong, J., Wang, C., Lai, W., et al. (2017b). Derivation of pluripotent stem cells with in vivo embryonic and extraembryonic potency. Cell *169*, 243–257.e225.

Zhang, H., Gayen, S., Xiong, J., Zhou, B., Shanmugam, A.K., Sun, Y., Karatas, H., Liu, L., Rao, R.C., Wang, S., et al. (2016). MLL1 inhibition reprograms epiblast stem cells to naive pluripotency. Cell Stem Cell *18*, 481–494.

Zhang, M., Wang, F., Kou, Z., Zhang, Y., and Gao, S. (2009). Defective chromatin structure in somatic cell cloned mouse embryos. J. Biol. Chem. *284*, 24981–24987.

Zhou, W., Choi, M., Margineantu, D., Margaretha, L., Hesson, J., Cavanaugh, C., Blau, C.A., Horwitz, M.S., Hockenbery, D., Ware, C., et al. (2012). HIF1α induced switch from bivalent to exclusively glycolytic metabolism during ESC-to-EpiSC/hESC transition. EMBO J. *31*, 2103–2116.

Stem Cell Reports, Volume *16*

Supplemental Information

Derivation of feeder-free human extended pluripotent stem cells

Ran Zheng, Ting Geng, Dan-Ya Wu, Tianzhe Zhang, Hai-Nan He, Hai-Ning Du, Donghui Zhang, Yi-Liang Miao, and Wei Jiang

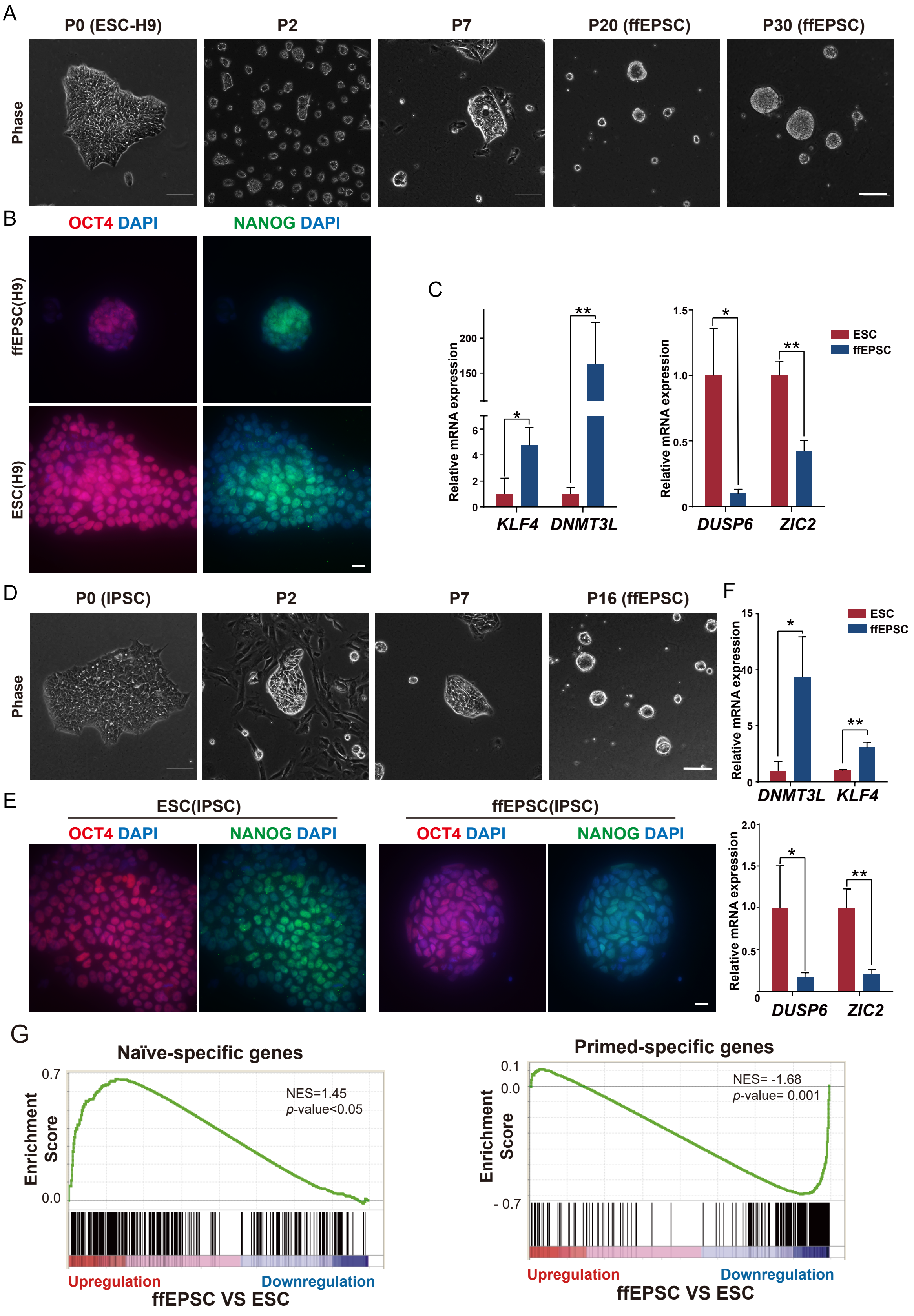

A
P0 (ESC-H9)
P2
P7
P20 (ffEPSC)
P30 (ffEPSC)
Phase
B
OCT4 DAPI
NANOG DAPI
ffEPSC(H9)
ESC(H9)
C
Relative mRNA expression
KLF4
DNMT3L
DUSP6
ZIC2
ESC
ffEPSC
D
P0 (IPSC)
P2
P7
P16 (ffEPSC)
Phase
E
ESC(IPSC)
ffEPSC(IPSC)
OCT4 DAPI
NANOG DAPI
F
Relative mRNA expression
DNMT3L
KLF4
DUSP6
ZIC2
ESC
ffEPSC
G
Naïve-specific genes
Enrichment Score
NES=1.45
p-value<0.05
Upregulation
Downregulation
ffEPSC VS ESC
Primed-specific genes
Enrichment Score
NES= -1.68
p-value= 0.001
Upregulation
Downregulation
ffEPSC VS ESC

Figure S1. Generation of human ffEPSCs from H9 or iPSCs under feeder-free condition.

A. The morphology of cells during the transition of ESC H9 into ffEPSCs. Scale bars = 100 μm.
B. H9-derived ffEPSCs showed positive staining of NANOG and OCT4. Scale bars = 20 μm.
C. Expression patterns of pre-implantation genes and post-implantation genes in ffEPSCs compared to H9-ESCs (three independent experiments).
D. The morphology of cells during the transition of human iPSCs into ffEPSCs. Scale bars = 100 μm.
E. human iPSC-derived ffEPSCs showed positive staining of NANOG and OCT4. Scale bars = 20 μm.
F. Expression patterns of pre-implantation genes and post-implantation genes in ffEPSCs compared to iPSCs (three independent experiments)
G. GSEA analysis showed the expression pattern of naive-specific genes and primed-specific genes in human ffEPSCs and ESCs.

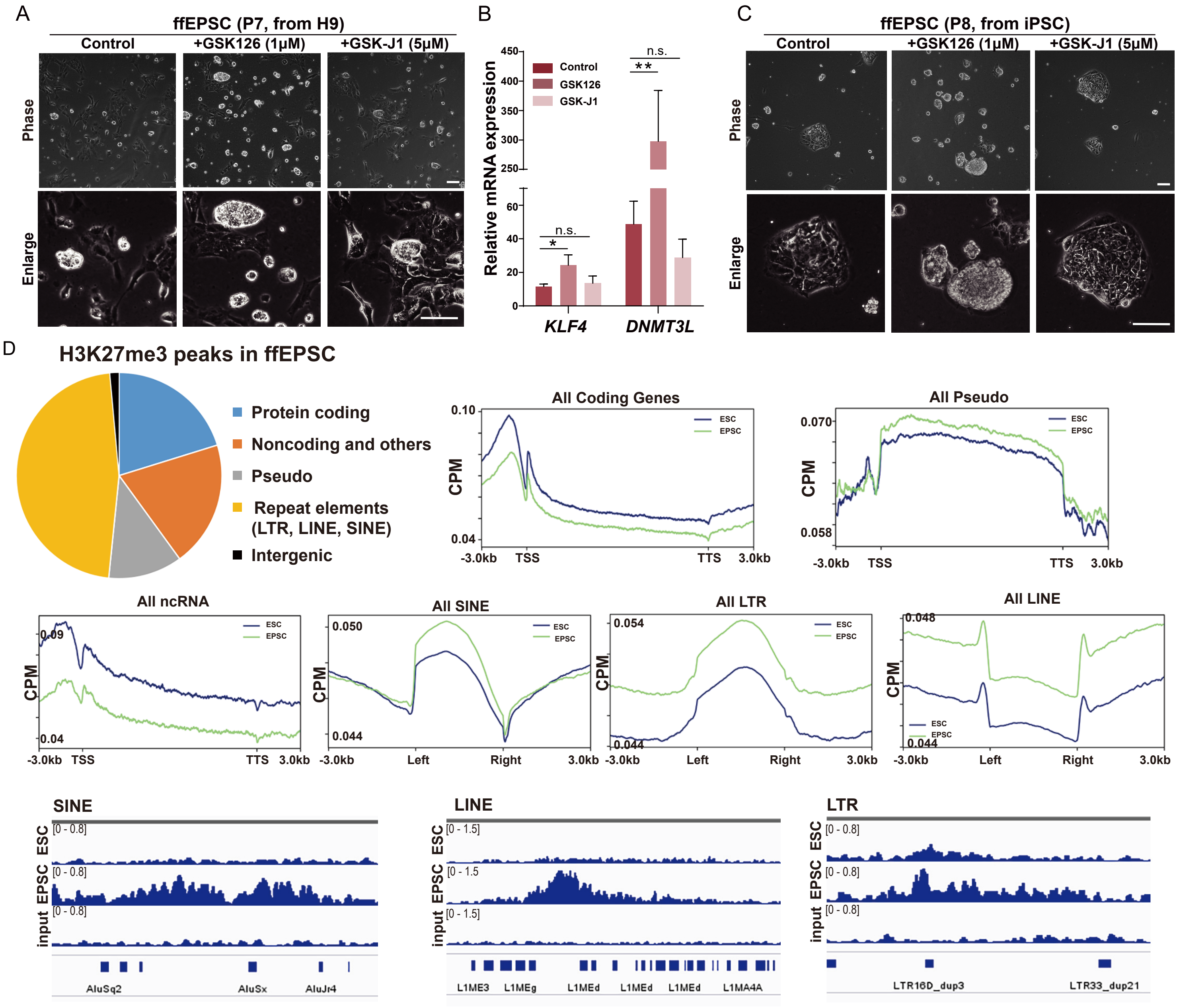
A
ffEPSC (P7, from H9)
Control
+GSK126 (1μM)
+GSK-J1 (5μM)
Phase
Enlarge
B
Relative mRNA expression
Control
GSK126
GSK-J1
n.s.
*
n.s.
**
KLF4
DNMT3L
C
ffEPSC (P8, from iPSC)
Control
+GSK126 (1μM)
+GSK-J1 (5μM)
Phase
Enlarge
D
H3K27me3 peaks in ffEPSC
Protein coding
Noncoding and others
Pseudo
Repeat elements (LTR, LINE, SINE)
Intergenic
All Coding Genes
All Pseudo
All ncRNA
All SINE
All LTR
All LINE
CPM
ESC
EPSC
-3.0kb
TSS
TTS
3.0kb
Left
Right
SINE
LINE
LTR
ESC
EPSC
input
[0 - 0.8]
[0 - 1.5]
AluSq2
AluSx
AluJr4
L1ME3
L1MEg
L1MEd
L1MA4A
LTR16D_dup3
LTR33_dup21

Figure S2. GSK126 facilitated iPSC-derived human ffEPSC transition.

A, Morphology of H9-derived ffEPSCs cultured with different chemical inhibitors GSK126 and GSK-J1. Scale bars = 100 μm.

B, Gene expression levels of ffEPSCs cultured with GSK-126, GSK-J1 or vehicle control (three independent experiments).

C. Morphology of iPSC-derived ffEPSCs cultured with different chemical inhibitors GSK126 and GSK-J1. Scale bars = 100 μm.

D. Aggregated genomic profiles of H3K27me3 ChIP-Seq around the pcGENE, psGENE, ncRNA, LINE, SINE, LTR and peak distribution.

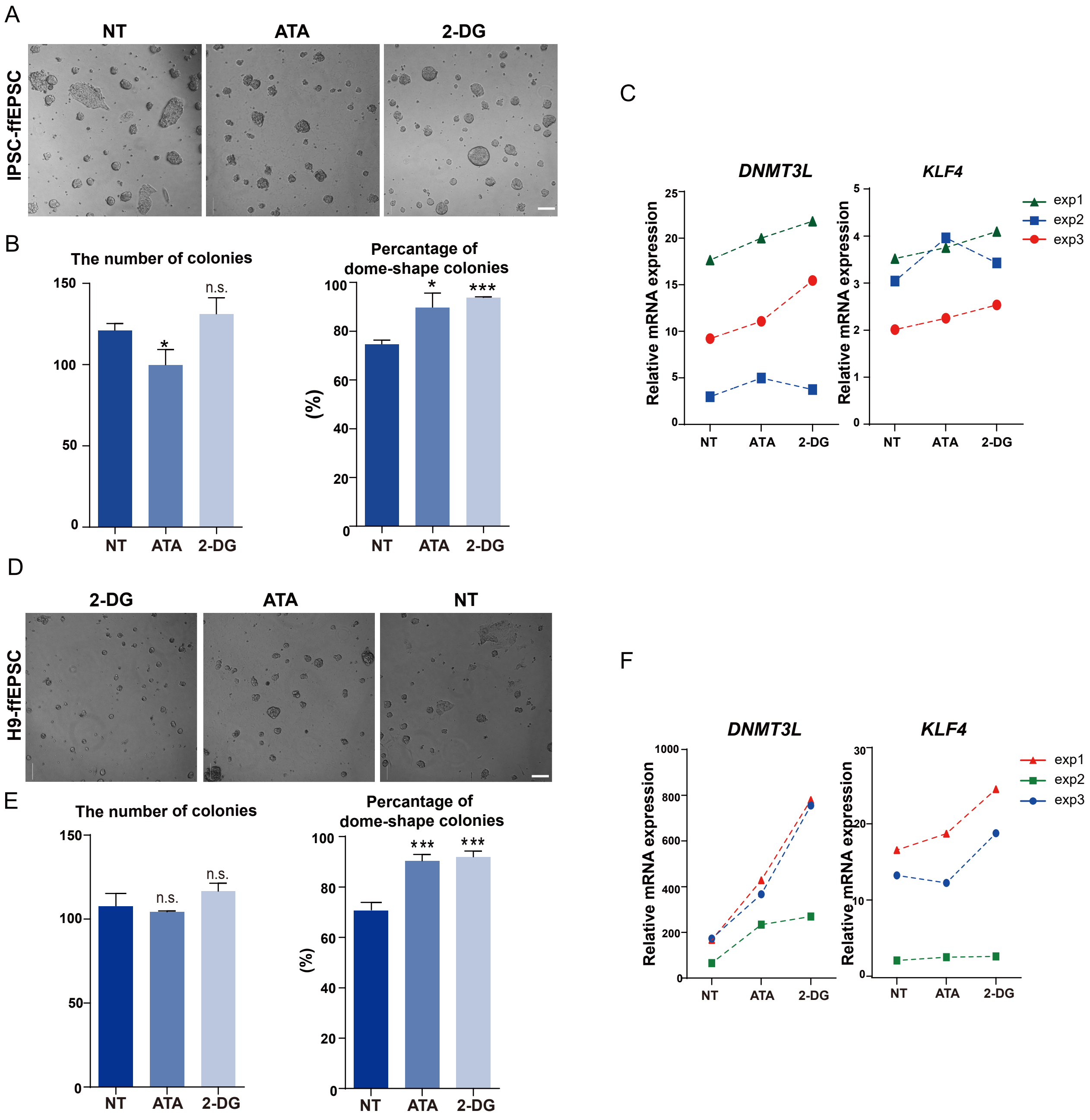
A
NT
ATA
2-DG
IPSC-ffEPSC
B
The number of colonies
n.s.
Percantage of
dome-shape colonies
(%)
C
DNMT3L
KLF4
Relative mRNA expression
exp1
exp2
exp3
D
H9-ffEPSC
E
F

Figure S3. Glycolysis inhibitor 2-DG and ATA facilitated H9 or iPSC-derived human ffEPSC maintenance.

A. Morphology of H9-derived ffEPSCs cultured with different chemical inhibitors 2-DG and ATA. Scale bars = 100 μm.

B. quantitative number and percentage of dome-shaped colonies of H9-derived ffEPSCs cultured with different chemical inhibitors 2-DG and ATA (three independent experiments).

C, RT-qPCR analysis of pre-implantation genes after treatment with glycolytic inhibitors. Results from three independent experiments were shown.

D. Morphology of iPSC-derived ffEPSCs cultured with different chemical inhibitors 2-DG and ATA. Scale bars = 100 μm.

E. quantitative number and percentage of dome-shaped colonies of iPSC-derived ffEPSCs cultured with different chemical inhibitors 2-DG and ATA (three independent experiments).

F, RT-qPCR analysis of pre-implantation genes after treatment with glycolytic inhibitors. Results from three independent experiments were shown.

Supplemental information：

Table S1: RNA-seq data of EPSCs and ESCs

Experimental Procedures

Immunofluorescence staining

Cells were grown on 24-well plates and washed by DPBS before fixed with 4% paraformaldehyde for 20 minutes at room temperature. Fixed cells were blocked with PBS containing 10% donkey serum and 0.3% Triton X-100 for 2 hours at room temperature. Then cells were incubated in the blocking buffer with diluted primary antibodies at 4°C overnight or at room temperature for 2 hours. The cells were further incubated in the blocking buffer with diluted second antibodies at room temperature for 2 hours after three times' washing with DPBS. Nuclei were counterstained with DAPI (1:10000, Thermo-Fisher).

Mouse embryos were collected to 96-well plates with a round bottom, fixed with 4% paraformaldehyde for overnight at 4°C, and then permeated in PBS containing 0.5% Triton X-100 for 30 minutes and blocked with 10% donkey serum in PBS containing 0.3% Triton X-100 for 2 hours at room temperature. Three times' washing with DPBS containing 0.01% Triton X-100 and 0.1% tween-20 was applied before every step. Embryos were incubated in the blocking buffer with diluted primary antibodies at 4°C overnight, and then further incubated with diluted second antibodies at room temperature for 1 hour after washing. At last, embryos were put in mounting medium with DAPI for image capture under the Zeiss LSM880 microscope.

The antibodies used were listed as follows: Anti-NANOG mouse IgG (SantaCruz, #SC-293121, 1:200), Anti-OCT4 mouse IgG (SantaCruz, #SC-5279, 1:200), Anti-OCT4 rabbit IgG (Cell Signaling Technologies, #2750, 1:200), Anti-GATA3 rabbit IgG (Cell Signaling Technologies, #5852, 1:200), Anti-GATA6 rabbit IgG (Cell Signaling Technologies, #5851, 1:200), Anti-OCT3/4 rabbit IgG (BD, #611203, 1:200,). Anti-aromatase rabbit IgG (ABclonal, #A12684, 1:100), Anti-MCT1 rabbit IgG (ABclonal, #A3013, 1:100), Anti-CDX2 rabbit IgG (ZSGB-bio, #ZA0520, 1:200), Donkey-Anti-Mouse-TRITC (Jackson immuno Research, #715-025-150, 1:200), Donkey-Anti-

Mouse-488 (Jackson immuno Research, #715-545-150, 1:200), Donkey-Anti-Rabbit-TRITC (Jackson immuno Research, #711-025-152, 1:200), Donkey-Anti-Rabbit-FITC (Jackson immuno Research, #711-095-152, 1:200).

Flow cytometry

Human ESCs were dissociated with accutase and ffEPSCs were dissociated with TrypLE, to generate single-cell suspension. Intracellular flow cytometry was operated with the Transcription Factor Buffer Set (BD Biosciences). Cells were stained with primary and then secondary antibodies diluted with 2% FBS in PBS containing 0.3% Triton X-100. Data were collected on a FACS Celesta flow cytometer (Becton Dickinson) and analyzed using FlowJo.

Western blot

Cell numbers were counted. Cells were lysed with 1X loading buffer, boiled for 10 minutes at 100°C, Proteins lysates were separated by 15% sodium dodecyl sulfate–poly-acrylamide gel electrophoresis and transferred onto a nitrocellulose membrane (Millipore). The membrane was blocked with 5% BSA in TBS buffer with 0.1% Tween-20 and incubated with antibody against H3K27me3 (1:1000, Cell Signaling Technology, #9733S), H3K4me3 (1:1000, Abclonal, #WH102597), H3K36me3 (1:3000, ABclonal, #A2366), H3K36me2 (1:3000, ABclonal, #A2365), H3K36me (1:3000, ABclonal, #A2364), H3K79me2 (1:3000, ABclonal, #A2368), H3K36Ac (1:500, ABclonal, #A16077) or H3 (1:3000, Proteintech, #17168-1-AP) overnight at 4°C. Then, the membrane was washed and incubated with secondary antibodies at room temperature for 2 hours. After washing, the membrane was visualized with SuperSignal® West Pico Chemiluminescent Substrate (ThermoFisher).

RT-qPCR

Total RNA was extracted with the HiPure Total RNA Mini Kit (Magen). 1-2 μg of total RNA was reversely transcribed into complementary DNA with 5×qRT super Mix.

qPCR was done in duplicated with 2×SYBR Green qPCR Master Mix (Bio-Rad). Glyceraldehyde-3-phosphate dehydrogenase (GAPDH) was used as an endogenous housekeeping control. Student's t-test (two-tailed, equal variance) was performed to obtain p-values for RT-qPCR experiments. Primer sequences are listed as follows: *NANOG* (CCCCAGCCTTTACTCTTCCTA, CCAGGTTGAATTGTTCCAGGTC); *OCT4* (CAAAGCAGAAACCCTCGTGC, TCTCACTCGGTTCTCGATACTG); *KLF4* (ACCCACACAGGTGAGAAACC, ATGCTCGGTCGCATTTTTGG); *DNMT3L* (CGCCCCATGTAAGGACAAGT, ATCGGGTGCAATCAGGGTTT); *ZIC2* (GCACGTCCACACCTCCGATAA, TGGACCTTCATGTGCTTCCGCAG); *DUSP6* (TGGAACGAGAATACGGGCG, CTTACTGAAGCCACCTTCCA); *EZH1* (CGGCTCGGGATGGAGGATTAC, GCCTTTGCACCCATATTTGCC); *EZH2* (GCTTCCTACATCGTAAGTGCAA, GCTCCCTCCAAATGCTGGTA); *KDM6A* (TGAATCCTGCAACCAGCCTC, AGGTTACACAACTGAGCCTGTA); *KDM6B* (CACCCACTGTGGTCTGTTGT, CGCCTCAGTAACAGCCAGAT); *GAPDH* (AATGAAGGGGTCATTGATGG, AAGGTGAAGGTCGGAGTCAA). Human-specific mitochondrial element (CGGGAGCTCTCCATGCATTT, GACAGATACTGCGACATAGGGT); Human-mouse conserved mitochondrial element (GCTAAGACCCAAACTGGGATT, GGTTTGCTGAAGATGGCGGTA).

Next generation sequencing and data analysis

Total RNA of human ESCs and ffEPSCs were prepared in duplicate with the HiPure Total RNA Mini Kit (Magen). Samples were sequenced on Illumina HiSeq X Ten PE150 at Annoroad Gene Technology Co. Ltd.

Sequencing reads were aligned to the human genome build hg38/GRCh38 with the HISAT2 (Pertea et al., 2016). Raw counts were performed with FeatureCounts (Liao et al., 2013) using GENCODE v29 human gene annotation (Harrow et al., 2012). Raw counts were normalized for total read counts using the size factors computed by the Bioconductor package DESeq2 (Love et al., 2014). Differential expression analysis was performed using the default settings of DESeq2 with p-value of <0.05 and filtering out

genes with TPM less than 1. To generate the heatmap for differential gene expression, TPM values were scaled relative to the mean expression of each gene across all samples in R (http://www.r-project.org/). The Gene Expression Omnibus (GEO) accession number for the RNA-seq raw data reported in this work is GSE137208 and expression values were shown as TPM in Table S1. Functional annotation of significantly different transcripts and enrichment analysis were performed with Clusterprofiler (Yu et al., 2012).

To quantify the specific genes for zygotic genome activation stage, two RNA-seq datasets of human early embryo development (GSE44183 (Xue et al., 2013) and GSE36552 (Yan et al., 2013)) were aligned to the human genome with the HISAT2 aligner, and raw counts were normalized to TPM and filtering out genes with TPM less than 20. The ZGA-specificity score of each transcript was defined as follows: Score = meanA - (meanOther+2*sdOther). Where meanA is the mean expression of the samples in certain stage, and meanOther and sdOther are the mean and SD of the expression levels in the other samples, respectively. Therefore, a positive score indicated that the gene was expressed in a certain stage at a considerably higher level than in the rest of the stages. A gene with a score of >0.5 was considered as specifically expressed in a certain stage.

For Gene Set Enrichment Analysis (GSEA) we used normalized counts by DEseq2 as input. The ZGA specific genes were generated by the overlap of two RNA-seq datasets (GSE44183 and GSE36552). The H3K27me3 ChIP-seq data was downloaded from GEO database (GSE89303) and accordingly analyzed (Yang et al., 2017).

Seahorse cellular flux assays

XF24 Cell Culture Microplates were pre-coated with Matrigel 2 hours before cell seeding. Human ESCs and EPSCs were seeded onto Matrigel-coated plate and cultured for 6 hours. Then the culture media was changed by base media (unbuffered DMEM supplemented with 2 mM Glutamine, pH 7.3-7.4) 500 μl per well about 1 hour before

the assay. Selective chemical inhibitors with proper concentrations were added during the measurements. Cell glycolysis stress test were measured using an XF24 Extracellular Flux Analyzer. All the data were normalized to DNA concentration calibrated by CyQuant™ Cell Proliferation Assay kit (Thermofisher).

REFERENCES:

Harrow, J., Frankish, A., Gonzalez, J.M., Tapanari, E., Diekhans, M., Kokocinski, F., Aken, B.L., Barrell, D., Zadissa, A., Searle, S*., et al.* (2012). GENCODE: the reference human genome annotation for The ENCODE Project. Genome research *22*, 1760-1774.

Liao, Y., Smyth, G.K., and Shi, W. (2013). featureCounts: an efficient general purpose program for assigning sequence reads to genomic features. Bioinformatics *30*, 923-930.

Love, M.I., Huber, W., and Anders, S. (2014). Moderated estimation of fold change and dispersion for RNA-seq data with DESeq2. Genome Biology *15*, 550.

Pertea, M., Kim, D., Pertea, G.M., Leek, J.T., and Salzberg, S.L. (2016). Transcript-level expression analysis of RNA-seq experiments with HISAT, StringTie and Ballgown. Nature Protocols *11*, 1650-1667.

Xue, Z., Huang, K., Cai, C., Cai, L., Jiang, C.-y., Feng, Y., Liu, Z., Zeng, Q., Cheng, L., Sun, Y.E*., et al.* (2013). Genetic programs in human and mouse early embryos revealed by single-cell RNA sequencing. Nature *500*, 593-597.

Yan, L., Yang, M., Guo, H., Yang, L., Wu, J., Li, R., Liu, P., Lian, Y., Zheng, X., Yan, J*., et al.* (2013). Single-cell RNA-Seq profiling of human preimplantation embryos and embryonic stem cells. Nature Structural & Molecular Biology *20*, 1131-1139.

Yang, Y., Liu, B., Xu, J., Wang, J., Wu, J., Shi, C., Xu, Y., Dong, J., Wang, C., Lai, W*., et al.* (2017). Derivation of Pluripotent Stem Cells with In Vivo Embryonic and Extraembryonic Potency. Cell *169*, 243-257.e225.

Yu, G., Wang, L.-G., Han, Y., and He, Q.-Y. (2012). clusterProfiler: an R Package for Comparing Biological Themes Among Gene Clusters. OMICS: A Journal of Integrative Biology *16*, 284-287.
